# The PPTC7/BNIP3/NIX axis induces cGAS/STING-mediated senescence and augments CAR-T efficacy by repressing tumor-intrinsic mitophagy

**DOI:** 10.1186/s13046-026-03713-7

**Published:** 2026-04-18

**Authors:** Kuai Yu, Xinxin Xiong, Bo Lu, Yaqun Wang, Tiantian Sun, Mei Xie, Xiaojun Xu, Yuanbin Song, Wei Xiao

**Affiliations:** 1 Department of Blood Transfusion, Key Laboratory of Jiangxi Province for Transfusion Medicine, The First Affiliated Hospital, Jiangxi Medical College, Nanchang University, Nanchang, Jiangxi 330209 China; 2 Guangdong Provincial Key Laboratory of Allergy & Clinical Immunology, The Second Affiliated Hospital of Guangzhou Medical University, Guangzhou, 510260 China; 3 Department of Hematology, The Seventh Affiliated Hospital, Sun Yat-sen University, 628 Zhenyuan Road, Guangming District, Shenzhen, Guangdong 518107 China; 4 Department of Hematologic Oncology, State Key Laboratory of Oncology in South China, Guangdong Provincial Clinical Research Center for Cancer, Sun Yat-sen University Cancer Center, No. 651 Dongfeng East Road, Yuexiu District, Guangzhou City, Guangdong Province China

**Keywords:** Multiple Myeloma, Mitophagy, Protein Phosphatase Targeting Cofactor 7, BNIP3/NIX, CGAS/STING Pathway, Chimeric Antigen Receptor T Cell

## Abstract

In multiple myeloma (MM), resistance to B-cell maturation antigen (BCMA)-targeted chimeric antigen receptor T-cell (CAR-T) treatment continues to limit therapeutic benefit, whereas the tumor-intrinsic factors governing sensitivity to CAR-T remain poorly understood. Here, integrating public single-cell RNA-seq data with transcriptomic profiling of MM cells before and after CAR-T exposure, we identified the mitochondrial protein PPTC7 as a key modulator linking tumor mitophagy to innate immune activation and CAR-T efficacy. Mechanistically, PPTC7 acted as a negative regulator of tumor-intrinsic mitophagy by promoting SCF^FBXL4-dependent ubiquitination and proteasomal degradation of the mitophagy receptors BNIP3 and NIX, thereby suppressing mitophagic flux. Consistent with this, PPTC7 overexpression inhibited mitophagy, caused mitochondrial dysfunction, and increased cytosolic leakage of mitochondrial DNA (mtDNA), whereas PPTC7 knockdown stabilized BNIP3/NIX, enhanced mitophagy, and reduced cytosolic mtDNA. The accumulated cytosolic mtDNA consequent to mitophagy suppression activated the cGAS/STING pathway, induced a senescence-associated secretory phenotype, and remodeled the inflammatory tumor microenvironment. Functionally, PPTC7-mediated mitophagy repression markedly enhanced CAR-T cytotoxicity in vitro and promoted CAR-T infiltration and tumor control in vivo. Rescue experiments further supported this axis: restoring mitophagy via NIX attenuated mtDNA leakage and dampened cGAS/STING signaling and CAR-T activity, whereas re-activating cGAS reinstated inflammatory signaling and CAR-T effector function. Collectively, our findings define a tumor-intrinsic PPTC7-BNIP3/NIX-mtDNA-cGAS/STING circuit in which suppression of mitophagy increases tumor immunogenicity and sensitizes MM to CAR-T therapy, highlighting PPTC7 as a potential immune-sensitizing target to overcome CAR-T resistance.

**Supplementary Information:**

The online version contains supplementary material available at 10.1186/s13046-026-03713-7.

## Highlights

Our findings reveal the mechanism by which PPTC7 regulates mitophagy, influencing CAR-T efficacy.

We demonstrate that PPTC7 inhibits mitophagy in MM cells by promoting the ubiquitin-dependent degradation of BNIP3 and NIX.

Our findings reveal that PPTC7 activates the cGAS/STING signaling pathway, thereby inducing senescence in MM cells and enhancing antitumor immunity.

We show that inhibition of mitophagy via PPTC7 increases the sensitivity of MM cells to CAR-T treatment.

This study provides novel molecular insights and a potential therapeutic target for enhancing CAR-T efficacy in MM, with promising clinical implications.

**Graphical abstract:**

**Supplementary Information:**

The online version contains supplementary material available at 10.1186/s13046-026-03713-7.

## Graphical abstract

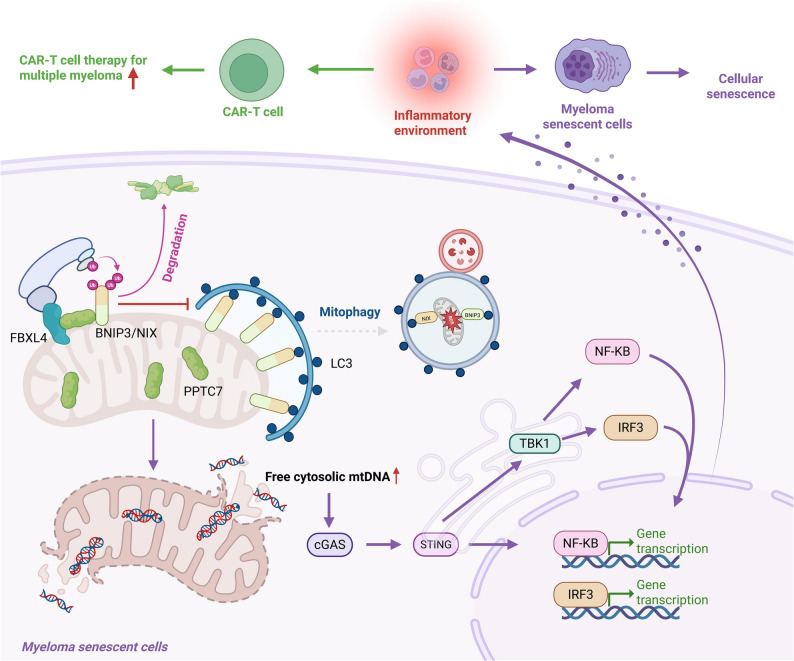

## Introduction

Multiple myeloma (MM) is a clonal plasma cell malignancy with substantial intertumoral heterogeneity and a recurrent clinical course [1]. Although therapeutic regimens based on proteasome inhibitors, immunomodulatory drugs, and monoclonal antibodies have improved patient management, disease relapse and treatment refractoriness remain common clinical challenges [2]. With the advancement of cellular immunotherapy, chimeric antigen receptor T cell (CAR-T) therapy targeting B-cell maturation antigen (BCMA) has shown remarkable progress in treating relapsed or refractory MM [3], with some patients achieving deep and sustained remission [4]. However, CAR-T therapy still faces significant challenges, including primary resistance, short-lived responses, and high relapse rates. One of the key underlying issues is the incomplete understanding of immune evasion mechanisms in tumor cells [5, 6]. Therefore, there is an urgent need to elucidate novel molecular mechanisms and identify critical regulators involved in CAR-T resistance in MM, in order to provide a theoretical basis and therapeutic targets for improving clinical outcomes.

As a targeted autophagic mechanism for mitochondrial quality control, mitophagy is crucial for both cellular homeostasis and the modulation of tumor immunity [7, 8]. Tumor cells may enhance mitophagy to eliminate damaged mitochondria [9], thereby preventing the release of mitochondrial DNA (mtDNA) into the cytosol. This suppression of cytosolic mtDNA release can inhibit activation of innate immune pathways such as the cGAS/STING axis, reducing tumor immunogenicity and enabling immune evasion [10, 11]. Previous studies have shown that activation of autophagic pathways during immunotherapy may impair antigen presentation, downregulate inflammatory cytokines, and weaken T cell activity, ultimately leading to therapeutic failure [12]. Consequently, modulating mitophagy has been proposed as a promising strategy to overcome immune resistance and improve responsiveness to immunotherapy. Nonetheless, whether mitophagy plays a central role in CAR-T resistance remains insufficiently explored.

PPTC7 (Protein Phosphatase Targeting Cofactor 7) is a recently identified mitochondrial protein that regulates mitophagy by promoting the ubiquitin-dependent degradation of autophagy receptors [13, 14]. On the other hand, the release of mtDNA activates the cGAS/STING pathway, leading to the induction of downstream interferons and pro-inflammatory cytokines, thereby enhancing antitumor immune responses, including the recruitment and activation of T cells [15]. STING agonist-induced IL-18 secretion has been shown to enhance CAR-T cell functionality [16]. Moreover, the cGAS/STING signaling pathway has been demonstrated to regulate both cellular senescence and inflammation, the latter being a known inducer of senescence. Activation of the cGAS-STING pathway can also target DNA damage responses to improve the efficacy of CD70-directed CAR-T cell therapy in renal carcinoma [17, 18]. Current evidence suggests that PPTC7 may serve as a critical link between mitophagy regulation and immune pathway activation [13]. Particularly within the tumor microenvironment, this mechanism may influence the sensitivity of tumor cells to CAR-T therapy and modulate their immune responsiveness [19, 20]. However, the role of PPTC7 in tumors has been rarely reported, and whether this pathway exerts functional significance in MM, as well as its impact on the immunotherapeutic efficacy of CAR-T cells, remains unclear and warrants further investigation.

The primary aim of this study was to elucidate the immunoregulatory role of PPTC7 in CAR-T therapy for MM, specifically whether PPTC7 enhances CAR-T efficacy by mediating the ubiquitin-dependent degradation of BNIP3/NIX, thereby suppressing mitophagy, promoting activation of the mtDNA-dependent cGAS/STING pathway, and inducing cellular senescence in MM cells. By focusing on the potential PPTC7-BNIP3/NIX-cGAS/STING regulatory axis, we sought to uncover a novel mechanism underlying immune resistance in MM. The findings from this study offer important theoretical insights for CAR-T therapy in MM and expand the landscape of immunotherapeutic targets, representing significant scientific value. Moreover, as a central node connecting mitophagy and inflammatory signaling, PPTC7 holds promising clinical potential as an immune-sensitizing strategy. Targeting PPTC7 may improve CAR-T response rates, prolong patient survival, and advance precision immunotherapy approaches for MM.

## Materials and methods

### Single-Cell RNA Sequencing (scRNA-seq) public data acquisition

To obtain scRNA-seq data related to MM and CAR-T therapy, we downloaded the GSE271915 dataset from the Gene Expression Omnibus (GEO) database (https://www.ncbi.nlm.nih.gov/geo/). This dataset was generated using the Illumina NovaSeq 6000 platform (Illumina, USA) and contains scRNA-seq data from MM samples collected before and after CAR-T therapy. We randomly selected paired bone marrow samples from five patients before and after BCMA-targeted CAR-T treatment. The samples were assigned to the Control group (GSM8388914, GSM8388916, GSM8388922, GSM8388924, and GSM8388926) and the Treat group (GSM8388913, GSM8388915, GSM8388921, GSM8388923, and GSM8388925).

### scRNA-seq analysis

Single-cell RNA-seq data were analyzed in R using the Seurat package. Cells meeting the criteria of 200 < nFeature_RNA < 5000 and percent.mt < 10 were retained for quality control. The top 2,000 variable genes were identified based on variance. Principal component analysis (PCA) was then performed for dimensionality reduction, and the first 20 principal components were selected according to the ElbowPlot results for subsequent analyses. Cell clusters were identified using the FindClusters function with the default resolution parameter (res = 0.5), followed by visualization with uniform manifold approximation and projection (UMAP).

Cell clusters were annotated manually according to known lineage markers from published studies, the CellMarker database (http://bio-bigdata.hrbmu.edu.cn/CellMarker/), and cluster-specific differentially expressed genes (DEGs) identified using the FindAllMarkers function in Seurat. To compare the relative abundance of each cell type before and after CAR-T therapy, we used the prop.table function in Seurat to calculate the proportion of each cell population in the Control and Treat groups. Visualization was performed using the ggplot2 package (version 3.4.0) to assess microenvironmental cellular composition changes induced by CAR-T treatment.

### Transcriptome sequencing and differential expression analysis

RPMI8226 MM cells were co-cultured with CAR-T cells at an effector-to-target ratio of 1:4 for 48 h. Cells were harvested before and after treatment, and total RNA was isolated with TRIzol reagent. RNA integrity was evaluated using an Agilent 2100 Bioanalyzer. Sequencing libraries were constructed with the NEBNext Ultra II RNA Library Prep Kit and sequenced on the Illumina NovaSeq 6000 platform to generate paired-end 150-bp reads.

After quality filtering with fastp (v0.23.2), clean reads were aligned to the human reference genome GRCh38 using STAR (v2.7.10a). Gene-level count matrices were generated with featureCounts (v2.0.3). Differential expression analysis was performed using DESeq2 (v1.38.3), with |log_2_FoldChange| > 1 and *P* < 0.05 defined as the criteria for significance. DEGs were visualized with the EnhancedVolcano package (v1.18.0).

### Gene ontology (GO) and kyoto encyclopedia of genes and genomes (KEGG) functional enrichment

DEGs were subjected to GO and KEGG pathway enrichment analyses using the clusterProfiler package v4.6.2 (Bioconductor, USA). All detected human genes were used as the background gene set, and annotations were based on the org.Hs.eg.db database. The GO analysis encompassed cellular component (CC), molecular function (MF), and biological process (BP) categories, while KEGG enrichment was based on its respective database (https://www.genome.jp/kegg/). Significance was defined as a Benjamini-Hochberg adjusted *p*-value of < 0.05. The ggplot2 package was applied to graph the enrichment outcomes. Using the VennDiagram package (v1.7.3) in R, the 50 most significant DEGs from the CAR-T treated group were cross-referenced with genes involved in autophagy-related pathways. For heatmap construction, the pheatmap package (v1.0.12, CRAN) was used, incorporating DESeq2-normalized counts subjected to Z-score scaling and hierarchical clustering based on genes.

### Cell line selection and in vitro culture

Human MM cell lines AMO1 (CRL-2972, ATCC, USA), RPMI8226 (CCL-155, ATCC, USA), U266 (TIB-196, ATCC, USA), NCI-H929 (CRL-9068, ATCC, USA), and human peripheral blood mononuclear cells (PBMCs; PCS-800-011, ATCC, USA) were grown in RPMI-1640 medium (11875093, Gibco, Thermo Fisher Scientific, USA). This medium was enriched with 10% fetal bovine serum (FBS; 10437028, Gibco, USA), 2 µmol/L glutamine (25030081, Gibco, USA), and 100 U/mL penicillin with 100 µg/mL streptomycin (15140122, Gibco, USA). HEK293T cells (CRL-3216, ATCC, USA) were maintained in DMEM (11965084, Thermo Fisher Scientific, USA) containing 10% FBS and 1% penicillin–streptomycin. Cells were cultured at 37 °C under 5% CO_2_ in a humidified incubator, with medium replacement performed routinely. Passaging was carried out every 2–3 d to maintain cell densities at 1 × 10^5^–1 × 10^6^ cells/mL. Adherent cells were dissociated with 0.25% trypsin–EDTA (25200056, Gibco, USA), neutralized with complete medium, centrifuged at 1,000 rpm for 5 min and resuspended in fresh medium. Mycoplasma contamination was routinely assessed using the MycoAlert Mycoplasma Detection Kit (LT07-703, Lonza, Switzerland) [21, 22].

### CAR-T preparation

PBMCs were incubated with CD3 magnetic beads (130-050-101, Miltenyi Biotec, Germany), and CD3^+^ T cells were isolated using magnetic column separation. CD3^+^ T cells were resuspended at 1 × 10^6^ cells/mL in ImmunoCult-XF T Cell Expansion Medium (10981, Stemcell Technologies, Canada) supplemented with IL-2 (200-02, PeproTech, USA) at 10 ng/mL. T cell activation was induced using ImmunoCult Human CD3/CD28 T Cell Activator (10971, Stemcell Technologies, Canada) at a final concentration of 25 µL/mL.

After 24 h of activation, T cells were transduced with a second-generation anti-BCMA CAR lentivirus (containing the 4-1BB costimulatory domain; Creative Biolabs, USA) in the presence of polybrene to enhance transduction efficiency. Cells were centrifuged at 180 × g for 1 min to facilitate infection. On day 3 of expansion, live cell counts were performed, and the culture volume was increased eightfold to continue incubation. On day 5, the culture volume was expanded at least fourfold. CAR-T was harvested on day 10 for subsequent experiments. All cell culture and expansion procedures were conducted at 37 °C in a humidified incubator with 5% CO_2_.

### Cell transfection and grouping

PPTC7, NIX, and cGAS overexpression constructs were generated by cloning their respective genes into the pLX307 lentiviral vector (632565, Clontech, USA). The resulting plasmids were co-transfected with packaging plasmids psPAX2 (12260, Addgene, USA) and pMD2.G (12259, Addgene, USA) into 293 T cells. For gene silencing, shRNA constructs targeting PPTC7, BNIP3, NIX, cGAS, and FBXL4 were cloned into the pLKO.1 vector (10878, Addgene, USA). These constructs were designated as sh-PPTC7#1 and sh-PPTC7#2, sh-BNIP3#1 and sh-BNIP3#2, sh-NIX#1 and sh-NIX#2, sh-cGAS#1 and sh-cGAS#2, and sh-FBXL4#1 and sh-FBXL4#2, with a scrambled sequence serving as the negative control (sh-NC). The targeting sequences of all shRNAs are listed in Table S1. Each overexpression or knockdown construct was co-transfected with psPAX2 and pMD2.G into HEK293T cells for lentiviral packaging. Viral supernatants were collected 48 h after transfection, passed through a 0.45 μm membrane filter (SLHV033RS, Millipore, USA) and used to infect AMO1 and RPMI8226 cells in the presence of polybrene (TR-1003, Sigma-Aldrich, USA) at 8 µg/mL. Transduced cells were selected with puromycin (A1113803, Gibco, USA) at 2 µg/mL for 7 days.

AMO1 and RPMI8226 cells were seeded in 6-well plates (3516, Corning, USA) at 1 × 10^5^ cells per well. When the cells reached 50%−60% confluency, lentiviral supernatant supplemented with 8 µg/mL polybrene (TR-1003, Sigma-Aldrich, USA) was added. Transduction was performed using a viral transduction reagent (K497500, Gibco, Thermo Fisher Scientific, USA) and maintained for 24 h.

The specific grouping was as follows: Group 1 (AMO1 and RPMI8226 cells): sh-NC, sh-PPTC7#1 and sh-PPTC7#2 (collectively referred to as sh-PPTC7), sh-FBXL4#1 and sh-FBXL4#2 (sh-FBXL4), and sh-PPTC7 + sh-FBXL4. Group 2 (AMO1 and RPMI8226 cells): sh-NC, sh-BNIP3#1, sh-BNIP3#2, sh-NIX#1, and sh-NIX#2. Group 3 (AMO1 cells): sh-NC, sh-PPTC7, sh-PPTC7 + sh-BNIP3, sh-PPTC7 + sh-NIX, and sh-PPTC7 + sh-BNIP3 + sh-NIX. Group 4 (AMO1 and RPMI8226 cells): vector, PPTC7, PPTC7 + NIX, and PPTC7 + NIX + c-GAS. Group 5 (AMO1 and RPMI8226 cells): Control, PPTC7, PPTC7 + rapamycin (RAPA) (autophagy inducer; AY22989, Selleck, China, 0.1 nmol/L for 72 h), and PPTC7 + ethidium bromide (EtBr; E1510, Sigma-Aldrich, USA, 400 ng/mL for 48 h). Group 6 (AMO1 and RPMI8226 cells): sh-NC, sh-c-GAS#1, and sh-c-GAS#2 (the more efficient sequence was used for subsequent experiments). Group 7 (AMO1 and RPMI8226 cells): sh-NC + vector, PPTC7 + sh-NC, and PPTC7 + sh-c-GAS.

### Co-culture of CAR-T with AMO1/RPMI8226 cells

To assess the effect of autophagy modulation on the cytotoxic activity of BCMA-targeted CAR-T cells, AMO1 and RPMI8226 cells were pretreated for 72 h with autophagy inhibitors or an autophagy inducer before co-culture. Cells were assigned to four groups: control group (DMSO, final concentration ≤ 0.1%, D2650, Sigma-Aldrich, USA), RAPA group (AY22989, Selleck, China, 0.1 nmol/L), SAR405 group (S7682, Selleck, China, 2 µmol/L), and autophinib group (S8596, Selleck, China, 0.1 µmol/L). Tumor cells were labeled with CFSE (565082, BD Biosciences, USA) at 1 µmol/L and then co-cultured with CAR-T cells at effector-to-target ratios of 0:1, 1:4, 1:2 and 1:1 for 24 h at 37 °C in 5% CO_2_.

For the lentiviral transduction groups, AMO1 and RPMI8226 cells were transduced with the indicated lentiviral constructs, labeled with CFSE, and then co-cultured with CAR-T at an E: T ratio of 1:4 for 24 h. After co-culture, cells were collected for subsequent analyses.

### Evaluation of CAR-T cytotoxicity

Following treatment and co-culture with CAR-T, AMO1 and RPMI8226 cells were stained with propidium iodide (PI; 421301, BioLegend, USA) at a final concentration of 0.2 µg/mL. Cells were collected, washed once with phosphate-buffered saline (PBS), and sequentially incubated with 5 µL FITC-Annexin V and 5 µL PI for 20 min at room temperature in the dark. Apoptosis was assessed by flow cytometry (FACS, BD Biosciences, USA) to evaluate CAR-T-mediated cytotoxicity. Granzyme B expression was analyzed by flow cytometry using a PE-conjugated anti-granzyme B antibody (372208, BioLegend, USA). Data were processed using FlowJo software to calculate the percentage of granzyme B-positive cells.

### Mitochondrial membrane potential (ΔΨm) detection

AMO1 and RPMI8226 cells were seeded in confocal dishes (353037, Corning, USA) at 1 × 10^5^ cells per well and cultured for 24 h until reaching 70%–80% confluence. After treatment, ΔΨm was assessed using a JC-1 ΔΨm Detection Kit (T3168, Thermo Fisher Scientific, USA). JC-1 working solution was added to the cells and incubated for 20 min at 37 °C in the dark. Cells were then washed twice with prewarmed PBS and maintained in fresh medium. Fluorescence signals were acquired with a confocal microscope (Leica TCS SP8, Leica Microsystems, Germany). Red fluorescence indicated JC-1 aggregates in mitochondria with intact membrane potential, whereas green fluorescence indicated JC-1 monomers in depolarized mitochondria. ΔΨm was further quantified by flow cytometry, and the proportion of cells with low ΔΨm was used for intergroup comparison.

### Reverse transcription quantitative polymerase chain reaction (RT-qPCR) analysis of mRNA expression

Total RNA was extracted from AMO1 and RPMI8226 cells using 1 mL TRIzol reagent (15596026, Thermo Fisher Scientific, USA) and incubated for 5 min at room temperature. Chloroform (0.2 mL; C2432, Sigma-Aldrich, USA) was then added, followed by thorough mixing and incubation for 2 min. Samples were centrifuged at 12,000 × g for 5 min at 4 °C, and the aqueous phase (~ 500 µL) was transferred to a fresh tube. RNA was precipitated with 500 µL isopropanol (RTC000079, Sigma-Aldrich, USA), incubated at − 20 °C for 6 min and then at room temperature for 10 min, pelleted by centrifugation at 12,000 × g for 10 min at 4 °C, washed with 75% ethanol, air-dried for 10 min and resuspended in RNase-free H2O. cDNA was synthesized using 5× All-In-One RT MasterMix (G490, Applied Biological Materials Inc., Canada/USA), followed by 20-fold dilution in RNase-free H2O. Quantitative PCR was performed with Taq Pro Universal SYBR qPCR Master Mix (Q712-03, Vazyme, China) on a CFX96 Touch Real-Time PCR Detection System (CFX96 Touch, Bio-Rad, USA) in triplicate. Relative mRNA expression was calculated using the 2^−ΔΔCt^ method with β-actin as the internal reference. Primer sequences are listed in Table S2.

### Assessment of mitophagy

AMO1 and RPMI8226 cells were seeded onto round glass coverslips pre-coated with poly-L-lysine (P8920, Sigma-Aldrich, USA) and allowed to adhere at 37 °C. After PBS washing, cells were fixed in 4% paraformaldehyde (P0099, Beyotime, China) at 4 °C overnight (12–16 h), blocked for 1 h at room temperature and incubated overnight at 4 °C with rabbit anti-LC3B antibody (#43566, 1:600, Cell Signaling Technology, USA). Alexa Fluor 488-conjugated goat anti-rabbit secondary antibody (ab150077, 1:1,000, Abcam, UK) was applied for 1 h at room temperature, followed by nuclear counterstaining with DAPI (#4083, Cell Signaling Technology, USA) for 10 min. Images were acquired using a confocal microscope (Dragonfly, Andor, Oxford, UK), and colocalization analysis was performed using NIS Elements software (Nikon Precision, China). LC3-positive puncta were quantified to evaluate autophagosome formation.

Mitophagy activity was further assessed using the mtKeima reporter plasmid (Addgene, #210338, USA). AMO1 and RPMI8226 cells from each treatment group were transfected by a liposome-mediated method, collected 48 h later, washed with PBS and resuspended for flow-cytometric analysis (BD Biosciences, USA). mtKeima fluorescence was measured at excitation wavelengths of 561 nm and 458 nm, and the 561/458 fluorescence ratio was used as an indicator of mitophagy activity.

To visualize mitophagy flux, the LAMP1-YFP plasmid (Addgene, #1817, USA) was co-transfected into mtKeima-expressing AMO1 and RPMI8226 cells using a liposome-mediated approach. After 48 h, cells were seeded into confocal imaging dishes and cultured for an additional 24 h. Confocal fluorescence microscopy was used to observe the colocalization of mtKeima and LAMP1-YFP signals. mtKeima was excited at 561 nm, and LAMP1-YFP at 488 nm, allowing for precise analysis of their spatial overlap.

### Detection of mtDNA release

AMO1 and RPMI8226 cells were seeded on glass coverslips and allowed to adhere at 37 °C. Mitochondria were labeled with MitoTracker Red (40741ES50, Yeasen, China) at a final concentration of 200 nM for 1 h, followed by incubation with PicoGreen dye (40753ES76, Yeasen, China) at 500 ng/mL for 15 min to label double-stranded DNA. Cells were then stained with Hoechst 33,342 working solution (HY-15559, MedChemExpress, USA) for 10 min with gentle agitation. After PBS washing, images were acquired using a Zeiss confocal microscope equipped with a 63× oil-immersion objective. To minimize interference from nuclear DNA, PicoGreen-positive nuclear signals were digitally excluded during image processing, and only cytoplasmic regions were retained for analysis. Cytoplasmic mtDNA release was quantified as the fluorescence intensity ratio of PicoGreen to MitoTracker Red using ImageJ software. Five random fields were analyzed for each image.

Cytoplasmic mtDNA was further quantified biochemically. Cytoplasmic and nuclear fractions were separated using a Cytoplasmic and Nuclear RNA Purification Kit (NGB-21000, Norgen Biotek, Canada). DNA from the cytoplasmic fraction was isolated with a DNA Extraction Kit (69506, Qiagen, Germany), and DNA concentration and purity were determined before downstream analysis. Cytoplasmic mtDNA abundance was measured by RT–qPCR, with nuclear DNA-specific primers used as negative controls to assess contamination. Primer sequences for mtDNA and nuclear DNA are listed in Table S2. Ct values from total cellular DNA were used for normalization, and relative cytoplasmic mtDNA levels were calculated using the 2^−ΔΔCt^ method. All reactions were performed in triplicate.

### Actinomycin D assay

AMO1 and RPMI8226 cells with lentiviral knockdown of PPTC7 or FBXL4 were treated with actinomycin D (66–81-9, Sigma-Aldrich, USA) at 10 µg/mL for 0, 3–6 h at 37 °C in 5% CO_2_. BNIP3 and NIX protein levels were then assessed by western blot (WB).

### Plasmid transfection and recombinant protein construction

To determine whether PPTC7 promotes FBXL4-dependent ubiquitination of BNIP3 and NIX, plasmids encoding HA-tagged ubiquitin (HA-Ub) and FLAG-tagged NIX or BNIP3 were generated. The HA-Ub sequence was cloned into pMT123 (18712, Addgene, USA). Full-length NIX and BNIP3 coding sequences were inserted into the FLAG-tagged pBICEP-CMV-3 vector (E7029, Sigma-Aldrich, USA). A catalytically inactive PPTC7 mutant (PPTC7-2 A; D105A and D223A) was cloned into pET28a-6HIS and expressed in Escherichia coli Rosetta (DE3) cells. Protein expression was induced with 0.3 mM IPTG at 18 °C, and recombinant protein was purified by Ni-NTA affinity chromatography. An FBXL4 mutant lacking the F-box domain (FBXL4-ΔF; Δ46–235) was cloned into pET28a-6HIS-MBP, expressed in Rosetta (DE3) cells and purified using the same procedure. For cellular assays, plasmid transfection was performed with Lipofectamine 3000 (L3000015, Thermo Fisher Scientific, USA). HA-Ub was co-transfected with FLAG-NIX or FLAG-BNIP3 into AMO1 and RPMI8226 cells under sh-FBXL4 conditions.

### Co-immunoprecipitation (Co-IP) assay for ubiquitination analysis

Forty-eight hours post-transfection, cells were incubated with the proteasome inhibitor MG132 (20 µM, S2619, Selleck, China) for 6 h to prevent degradation of ubiquitinated proteins. After lysis with RIPA buffer (89901, Thermo Fisher Scientific, USA), cell lysates were subjected to immunoprecipitation using anti-FLAG M2 agarose magnetic beads (M8823, Sigma-Aldrich, USA) to enrich FLAG-tagged NIX or BNIP3 proteins along with their ubiquitinated forms.

Following thorough washing of the immunoprecipitates, proteins were separated by SDS-PAGE and analyzed by WB. Anti-HA antibody (3724, Cell Signaling Technology, USA; 1:1000) and anti-FLAG antibody (F7425, Cell Signaling Technology, USA; 1:1000) were used to detect HA-Ub-modified NIX and BNIP3 and to assess the levels of FLAG-NIX or FLAG-BNIP3, respectively. β-actin was used as the loading control for total cell lysates.

### Assessment of cellular senescence

Cellular senescence was assessed by senescence-associated β-galactosidase (SA-β-gal) staining using a commercial kit (KAA002, Sigma-Aldrich, USA). Stained cells were examined under a light microscope, and the proportion of SA-β-gal-positive cells was calculated.

### WB analysis of protein expression

Transplanted tumor tissues from each group and cultured cells in the logarithmic growth phase were collected for protein extraction. Total protein was isolated using RIPA lysis buffer (89900, Thermo Fisher Scientific, USA). After lysis on ice for 30 min, samples were centrifuged at 12,000 rpm for 15 min at 4 °C, and the supernatants were collected. Protein concentrations were determined using the Pierce BCA Protein Assay Kit (23225, Thermo Fisher Scientific, USA). Equal amounts of protein were mixed with 5× SDS sample loading buffer (NP0007, Thermo Fisher Scientific, USA), boiled for 5 min and resolved by 10% SDS–PAGE. Proteins (40 µg per sample) were then transferred to PVDF membranes (IPVH00010, Millipore, USA). Membranes were blocked with 5% non-fat milk (1706404, Bio-Rad, USA) for 1 h at room temperature and incubated overnight at 4 °C with the following primary antibodies: anti-LC3 (12741, Cell Signaling Technology, USA; 1:1,000), anti-p62 (ab109012, Abcam, UK; 1:10,000), anti-PPTC7 (21884-1-AP, Proteintech, China; 1:5,000), anti-BNIP3 (ab109362, Abcam, USA; 1:1,000), anti-NIX (12396 S, Cell Signaling Technology, USA; 1:1,000), anti-FBXL4 (D163894-0025, Sangon Biotech, China; 1:1,000), anti-cGAS (79978, Cell Signaling Technology, USA; 1:1,000), anti-STING (13647, Cell Signaling Technology, USA; 1:1,000), anti-p-TBK1 (5483, Cell Signaling Technology, USA; 1:1,000), anti-p-IRF3 (4947, Cell Signaling Technology, USA; 1:1,000), anti-p-NF-κB (3033, Cell Signaling Technology, USA; 1:1,000), anti-p16 (80772, Cell Signaling Technology, USA; 1:1,000) and anti-p21 (64016, Cell Signaling Technology, USA; 1:1,000). After three washes in TBST (10 min each), membranes were incubated with HRP-conjugated goat anti-rabbit IgG secondary antibody (7074, Cell Signaling Technology, USA; 1:10,000) for 1 h at room temperature and washed again three times with TBST. Signals were detected using an enhanced chemiluminescence kit (32106, Thermo Fisher Scientific, USA) and imaged with the Gel Doc XR+ system (1708195, Bio-Rad, USA). Band intensities were quantified in ImageJ, with β-actin (4970, Cell Signaling Technology, USA; 1:1,000) as the loading control.

### Establishment and grouping of animal models

Female NOD SCID mice aged 4–5 weeks (Aniphe Biolaboratory, China) were maintained under specific pathogen-free conditions at 25 ± 2 °C with 40–60% humidity and a 12 h light/dark cycle, with free access to food and water. After 1 week of acclimatization, mice were used for MM model construction [23].

To generate a humanized immune system mouse model, 5 × 10^4^ CD34^+^ human hematopoietic stem cells (70012, STEMCELL Technologies, Canada) were injected via the tail vein. One to two weeks after injection, peripheral blood was collected to assess the reconstitution of human immune cells. Mice with > 45% hCD45^+^ cells in peripheral blood were considered successfully reconstituted. Tumor implantation was then performed [24].

Mice were randomly assigned to three groups: vector, PPTC7 and PPTC7 + NIX (*n* = 6 per group). RPMI8226 cells stably expressing the indicated constructs were harvested, and 1.0 × 10^6^ cells in 100 µL were injected subcutaneously into the corresponding mice. Tumor volume was measured weekly using a vernier caliper (500-159-30, Mitutoyo, Japan) and calculated as length × width^2 × 0.5. Mice were euthanized when tumor volume exceeded 1 cm^3^. Tumors were excised and divided into two portions: one was fixed in 4% paraformaldehyde (P0099-3L, Beyotime, China) for immunohistochemistry (IHC), and the other was snap-frozen in liquid nitrogen for subsequent experimental analyses [25, 26].

For CAR-T treatment studies, mice were assigned to five groups: control, CAR-T, CAR-T + PPTC7, CAR-T + PPTC7 + RAPA and CAR-T + PPTC7 + NIX (*n* = 6 per group). RPMI8226 cells (CCL-155, ATCC, USA) were transduced with a lentiviral vector encoding green fluorescent protein-luciferase (GFP-Luc) and purified by fluorescence-activated cell sorting (FACS, BD Biosciences, USA). A total of 1 × 10^6^ transduced cells were injected subcutaneously into the dorsal flank to establish xenograft tumors. Seven days later, 5 × 10^6^ BCMA CAR-T cells in 100 µL PBS were administered via tail-vein injection. RAPA (553211, Sigma-Aldrich, USA) was given intraperitoneally at 1 mg/kg once daily. Tumor burden was monitored weekly by bioluminescence imaging using the Lago X imaging system (Spectral Instruments Imaging, USA), and signals were quantified as average radiance using Living Image software (Amiview, USA). Four weeks after tumor-cell inoculation, mice were euthanized and tumors were excised, photographed and weighed [27].

### IHC staining

Tumor tissues were fixed in 4% paraformaldehyde for at least 48 h, embedded in paraffin and sectioned at 5 μm. Sections were dewaxed at 60 °C for 2 h, rehydrated through a graded ethanol series (100%, 95%, 80% and 70%) and rinsed with PBS (pH 7.4; 10010001, Thermo Fisher Scientific, USA). Antigen retrieval was performed in sodium citrate buffer (10000, Xabiolite, China) by boiling for 15 min. After cooling, endogenous peroxidase activity was quenched with 3% H2O2 (MM0750, Maokang Bio, China) for 8 min, followed by blocking with 10% goat serum (C0265, Beyotime, China) for 40 min at 24 °C. Sections were then incubated overnight at 4 °C with the following primary antibodies: anti-PPTC7 (ab122548, Abcam, UK; 1:100), anti-BNIP3 (ab109362, Abcam, USA; 1:100), anti-NIX (ab109414, Abcam, UK; 1:100), anti-CD3 (24581, Cell Signaling Technology, USA; 1:100), anti-CD4 (25229, Cell Signaling Technology, USA; 1:100), anti-CD8 (85336, Cell Signaling Technology, USA; 1:100) and anti-caspase 9 (ab32539, Abcam, UK; 1:100). After PBS washing, sections were incubated with HRP-conjugated goat anti-rabbit IgG (ab6702, Abcam, UK; 1:5,000) for 30–60 min at room temperature. Signals were developed with 3,3′-diaminobenzidine (DAB; CB0366173, Chemical Book, China) for 2 min and counterstained with hematoxylin (HY-N0116, MedChemExpress, China). Representative images were acquired using an inverted microscope (ECLIPSE Ti2, Nikon, Japan), and staining intensity was quantified with Image-Pro Plus v6.0.

### Transmission electron microscopy (TEM) for autophagosome formation

TEM (JEM-1400, JEOL, Japan) was employed to examine mitochondrial morphology and autophagic structures. Tumor tissues were fixed in 4% paraformaldehyde for 12 h, followed by dehydration for 24 h. The tissue blocks were subsequently fixed in 3% glutaraldehyde (T16427, Saint-bio, China). After cryosectioning on ice, autophagic structures involving mitochondria were visualized using TEM. Images were captured at a magnification of 6,000×.

### Detection of inflammatory cytokine levels

Levels of TNF-α, IL-6 and IL-1β in cell-culture supernatants were measured by enzyme-linked immunosorbent assay using commercial kits for TNF-α (CSB-E04740h, Cusabio, China), IL-6 (CSB-E04638h, Cusabio, China) and IL-1β (CSB-E08053h, Cusabio, China). Samples and corresponding detection reagents were added to 96-well plates and incubated for 1 h, followed by substrate development. Absorbance was measured using a microplate reader (BioTek, USA), and cytokine concentrations were calculated accordingly.

For tumor tissues, samples from each treatment group were minced and homogenized in tissue lysis buffer (P0013B, Beyotime, China). Homogenates were centrifuged at 13,500 rpm for 15 min at 4 °C, and the supernatants were collected for protein extraction. Protein concentrations were determined using a BCA assay kit (P0009, Beyotime, China). TNF-α, IL-6 and IL-1β levels were then quantified by ELISA using the same kits.

### Flow cytometry analysis of immune cell infiltration

Flow cytometry was used to assess the infiltration levels of CD3^+^, CD4^+^, and CD8^+^ immune cells in the tumor tissues of mice from each experimental group. Fresh tumors were dissociated into single-cell suspensions, and immune cells were enriched using Percoll (40501ES60, Yeasen, China). A total of 1 × 10^6^ cells were washed with 2 mL of PBS containing 1% FBS (C0235, Beyotime, China) and 0.2% EDTA (60126ES02, Yeasen, China), followed by centrifugation at 350 × g for 6 min. Cells were then incubated for 30 min with Fc receptor blocker (Magic™ Fc Receptor Blocker, CDN-ZF1, Abace-biology, China) and the following antibodies: FITC-conjugated anti-CD3 (1:200; 981002, BioLegend, USA), APC-conjugated anti-CD4 (1:100; 100412, BioLegend, USA), PerCP/Cyanine5.5-conjugated anti-CD8 (1:100; 980918, BioLegend, USA), and PE anti-BCMA (1:100; 357504, Biolegend, USA) [28]. After staining, cells were washed three times with PBS or staining buffer and analyzed on an LSRFortessa flow cytometer (BD Biosciences, USA).

### Statistical analysis

All experiments were independently repeated at least three times. Data are presented as mean ± standard deviation (SD). Statistical analyses were performed using GraphPad Prism 9.0 software (GraphPad Software, USA). Comparisons between two groups were conducted using a two-tailed unpaired Student’s t-test. For multiple group comparisons, one-way analysis of variance (ANOVA) followed by Dunnett’s post hoc test was applied. Two-way ANOVA was used when comparisons involved two or more independent variables. Statistical significance of data such as flow cytometry results, qPCR, and WB densitometry was determined using the methods described above. A *p*-value < 0.05 was considered statistically significant, with significance levels denoted as *p < 0.05, **p < 0.01, and ***p < 0.001.

## Results

### scRNA-seq and transcriptomic sequencing reveal the relationship between mitophagy and CAR-T therapy in MM

**Fig. 1:**
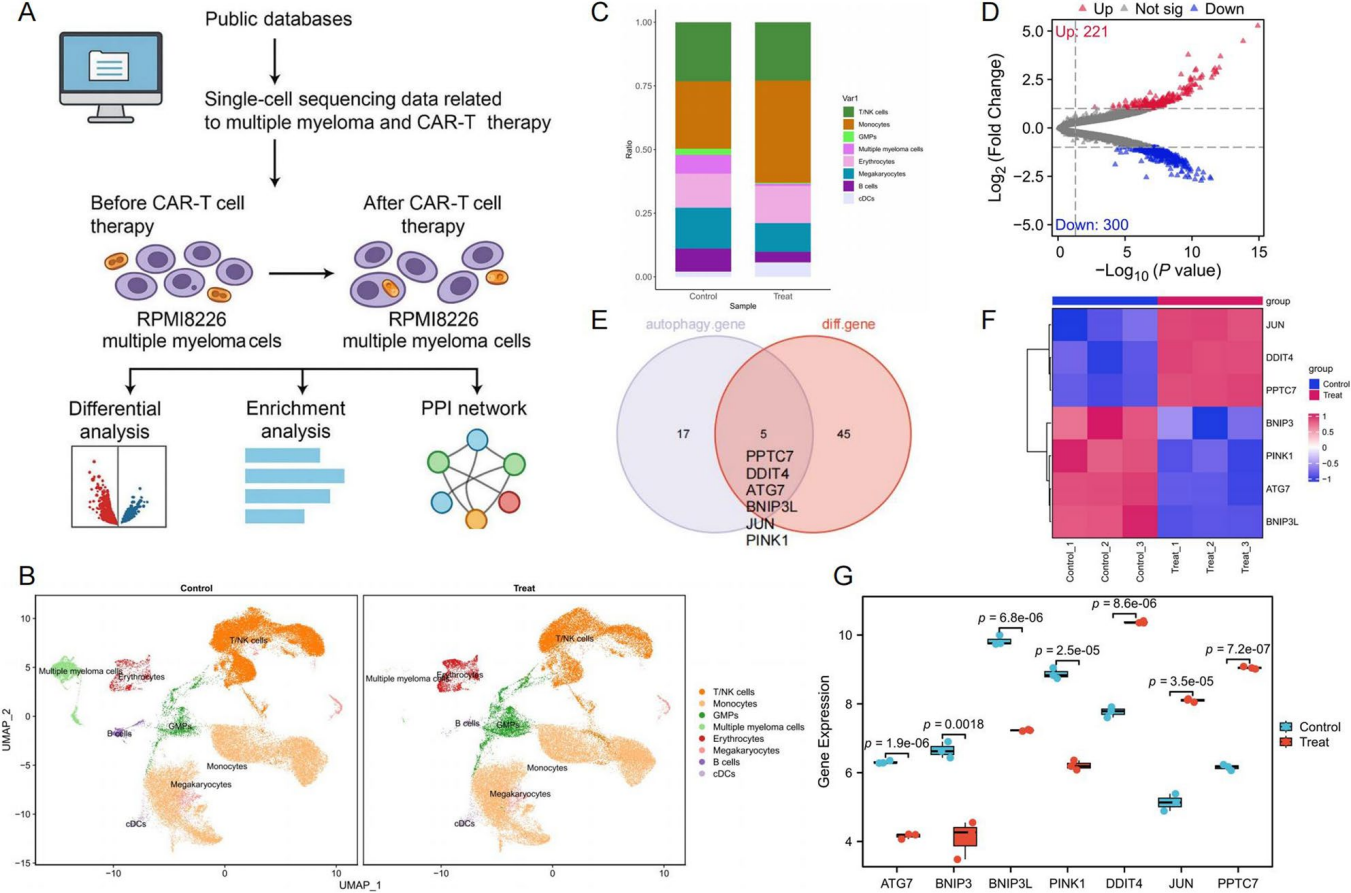
scRNA-seq and transcriptomic analysis reveal the expression patterns of mitophagy-related genes during CAR-T therapy in MM. Note: **A** Workflow of bioinformatic analysis integrating scRNA-seq data from public databases and transcriptomic profiles of RPMI8226 cells before and after CAR-T treatment to identify key genes associated with mitophagy; (**B**) UMAP plot illustrating the distribution of different cell types pre- and post-CAR-T treatment; (**C**) Bar chart depicting changes in cell population composition following CAR-T therapy; (**D**) Volcano plot showing DEGs in MM cells before and after CAR-T treatment; (**E**) Venn diagram displaying the intersection between DEGs and autophagy-related pathway genes, resulting in the identification of five mitophagy-related DEGs; (**F**-**G**) Heatmap and boxplot of autophagy-related DEGs across sample groups. GSE271915 dataset: Control = 5, Treat = 5; Transcriptome sequencing: Control = 3, Treat = 3

To investigate the role of mitochondria-mediated mitophagy in CAR-T therapy for MM, we first downloaded publicly available scRNA-seq datasets related to MM and CAR-T treatment. In addition, transcriptomic profiling was conducted on RPMI8226 MM cells collected before and after CAR-T therapy. Differential expression analysis, enrichment analysis, and PPI network construction were employed to elucidate the relationship between autophagy and the response of MM cells to CAR-T therapy (Fig. 1A). In the single-cell dataset GSE271915, 20 cell clusters were identified by unsupervised clustering. The UMAP projection showed a highly consistent distribution of cell clusters across pre- and post-treatment samples, confirming the robustness of cellular subtype classification (Figure S1A). Based on literature-curated lineage-specific marker genes and CellMarker annotations, a total of eight cell types were identified: T/NK cells, monocytes, granulocyte-macrophage progenitors (GMPs), erythrocytes, megakaryocytes, conventional dendritic cells (cDCs), MM cells, and B cells (Figure S1B and Fig. 1B). The cellular composition differed markedly between the CAR-T treatment and control groups. Quantitative analysis of cell-type proportions between the control and treatment groups revealed a reduced fraction of MM cells and increased proportions of monocytes and cDCs in the CAR-T group (Fig. 1C), suggesting that CAR-T therapy may induce remodeling of the MM cellular microenvironment.

To further identify key regulatory genes associated with CAR-T therapy, we performed differential expression analysis on MM cells before and after CAR-T treatment. The volcano plot revealed 221 upregulated and 300 downregulated genes in the treatment group (Fig. 1D). To characterize the biological significance of these DEGs, GO and KEGG enrichment analyses were conducted. GO analysis indicated that CAR-T-related DEGs were significantly enriched in pathways such as “cell cycle,” “chromosomal structure,” and “ribosome binding,” suggesting that CAR-T treatment may influence tumor cell proliferation and metabolic activity (Figure S2A). KEGG analysis further revealed significant enrichment in mitophagy, apoptosis, DNA repair and cellular senescence pathways, highlighting the involvement of mitophagy and stress-response programs in the antitumor effects of CAR-T therapy (Figure S2B).

The top 50 DEGs were intersected with genes enriched in autophagy and mitophagy pathways, resulting in the identification of five overlapping genes: PPTC7, DDIT4, ATG7, BNIP3L (NIX), JUN, and PINK1 (Fig. 1E). These genes are all closely associated with mitochondrial function, stress responses, and autophagy regulation. PPTC7, recently identified as a negative regulator of basal mitophagy, modulates the protein degradation of BNIP3 and NIX (BNIP3L), thereby influencing mitophagic activity [13]. Further expression heatmap analysis of these autophagy-related DEGs revealed that in the Treat group, DDIT4, PPTC7, and JUN were significantly upregulated, whereas ATG7, BNIP3L, BNIP3, and PINK1 were markedly downregulated. Among them, PPTC7 exhibited the most pronounced differential expression (Fig. 1F-G). These findings suggest that the PPTC7/NIX-BNIP3 axis is involved in modulating the mitophagy status of myeloma cells during CAR-T therapy.

### Inhibition of autophagy enhances the sensitivity of MM Cells to CAR-T therapy

**Fig. 2:**
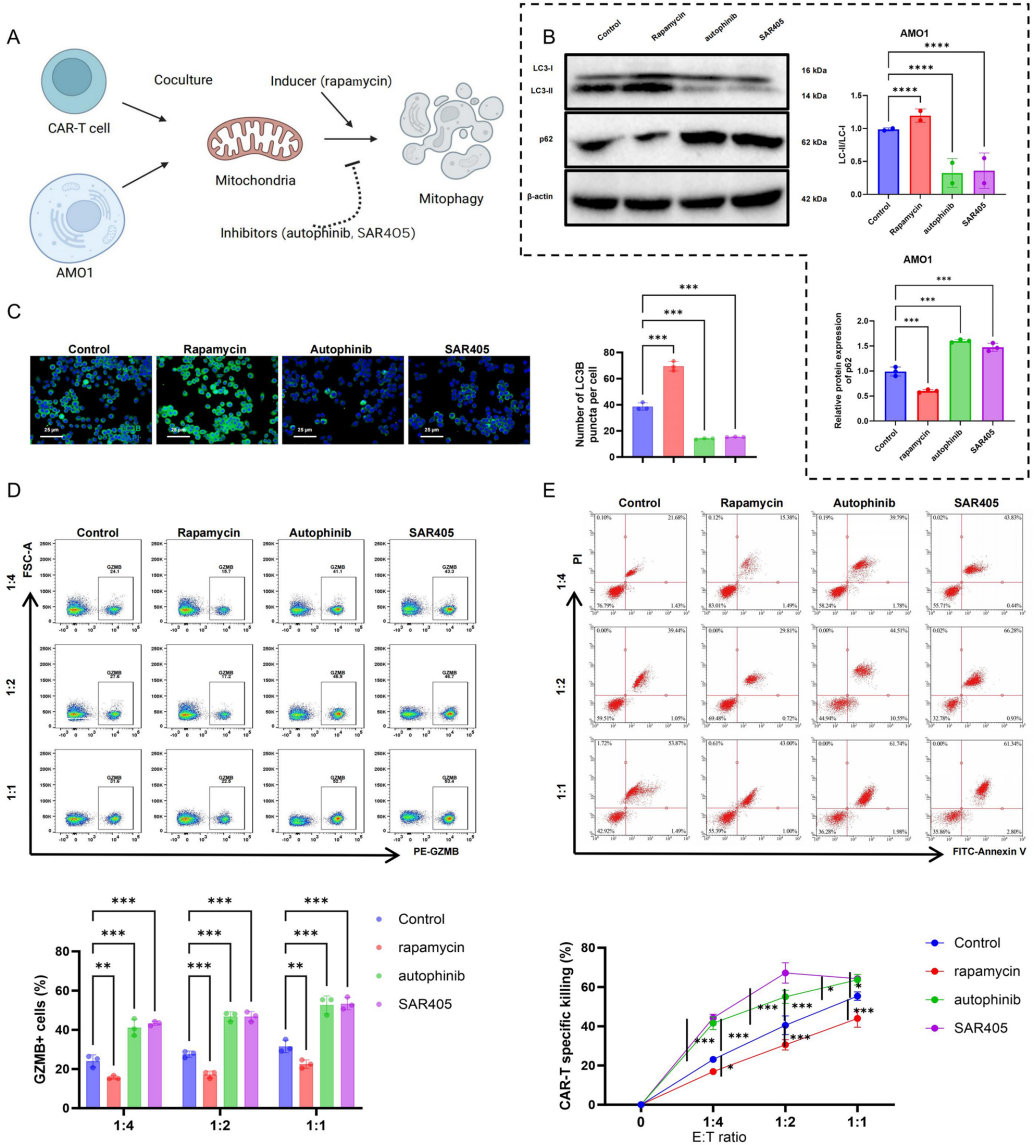
Autophagy inhibition enhances the sensitivity of MM AMO1 cells to CAR-T therapy.Note: **A** Schematic overview of the experimental design evaluating the impact of mitophagy inhibition on CAR-T treatment response, created using BioRender; (**B**) WB analysis of the LC3-II/LC3-I ratio and p62 accumulation in AMO1 cells to assess changes in autophagic activity; (**C**) Immunofluorescence staining to visualize autophagosome (LC3) formation in AMO1 cells (bar = 25 μm); (**D**) Flow cytometric analysis of granzyme B expression in CAR-T to evaluate cytotoxic activity; (**E**) Flow cytometric detection of apoptosis in AMO1 cells following CAR-T treatment. All cellular experiments were performed in triplicate. *p < 0.05, **p < 0.01, ***p < 0.001, ****p < 0.0001 indicate statistical significance between groups

To investigate whether autophagy influences MM-cell sensitivity to CAR-T therapy, AMO1 and RPMI8226 cells were treated with autophagy inhibitors or an autophagy inducer before co-culture with BCMA CAR-T cells (Fig. 2A). After 72 h of treatment, WB analysis showed that RAPA increased the LC3-II/LC3-I ratio and reduced p62 accumulation, consistent with enhanced autophagic activity. In contrast, autophinib and SAR405 decreased the LC3-II/LC3-I ratio and increased p62 levels, indicating inhibition of autophagic flux (Fig. 2B & Figure S3A). Immunofluorescence staining for LC3 further supported these findings. RAPA treatment markedly increased the number of LC3-positive puncta in both AMO1 and RPMI8226 cells, whereas autophinib- or SAR405-treated cells displayed fewer LC3-positive autophagosomes than control cells (Fig. 2C & Figure S3B).

Subsequently, AMO1 and RPMI-8226 cells subjected to the aforementioned treatments were co-cultured with BCMA CAR-T at various E: T ratios (1:4, 1:2, and 1:1) for 24 h. Granzyme B expression in CAR-T was assessed via flow cytometry. The results showed that, across all E: T ratios, RAPA-pretreate d AMO1 and RPMI-8226 cells significantly suppressed granzyme B expression in CAR-T compared with the control group, whereas pretreatment with autophinib or SAR405 led to a marked increase in granzyme B expression (Fig. 2D & Figure S3C). To further evaluate the cytotoxicity of CAR-T against AMO1 and RPMI-8226 cells, PI staining combined with flow cytometry was conducted. The analysis revealed that, at E: T ratios of 1:4, 1:2, and 1:1, RAPA pretreatment resulted in reduced apoptosis of AMO1 and RPMI-8226 cells compared with the control. In contrast, autophinib and SAR405 pretreatment significantly enhanced apoptosis in AMO1 cells (Fig. 2E & Figure S3D). This study also examined apoptosis in myeloma cells without co-culture with CAR-T cells. The results showed that autophagy inhibitors induced apoptosis in AMO1 and RPMI-8226 cells, whereas autophagy inducers had the opposite effect; however, the extent of these changes in apoptosis was markedly smaller compared with co-culture with CAR-T cells (Figure S4).

These results suggest that autophagy inhibition is a key determinant of MM-cell sensitivity to CAR-T therapy. Accordingly, targeting autophagy may enhance the antitumor efficacy of CAR-T treatment.

### Knockdown of PPTC7 stabilizes mitophagy receptors BNIP3 and NIX, leading to altered mitochondrial turnover in MM cells

To define the role of PPTC7 in mitophagy regulation in MM cells, PPTC7 protein expression was first examined in U266, AMO1, NCI-H929 and RPMI8226 cells, with human PBMCs used as a reference. PPTC7 expression was reduced in all four MM cell lines relative to PBMCs, with the lowest levels detected in AMO1 and RPMI8226 cells, which were selected for subsequent analyses (Figure S5A).

Using lentiviral transduction, PPTC7 was overexpressed in AMO1 and RPMI8226 cells to establish PPTC7-overexpression and vector control groups. WB analysis confirmed a marked upregulation of PPTC7 protein in the PPTC7-overexpression group compared with the vector group (Figure S5B). We next assessed autophagy by measuring the LC3-II/LC3-I ratio and p62 accumulation. In the PPTC7 group, LC3-II/LC3-I levels were significantly decreased, while p62 accumulation was markedly increased, indicating suppression of mitophagy (Figure S5C). Furthermore, JC-1 staining was used to evaluate ΔΨm. Both immunofluorescence staining and flow cytometry analysis demonstrated that ΔΨm was significantly reduced in the PPTC7 group compared with the vector group (Figure S5D-E), suggesting compromised mitochondrial function.

**Fig. 3:**
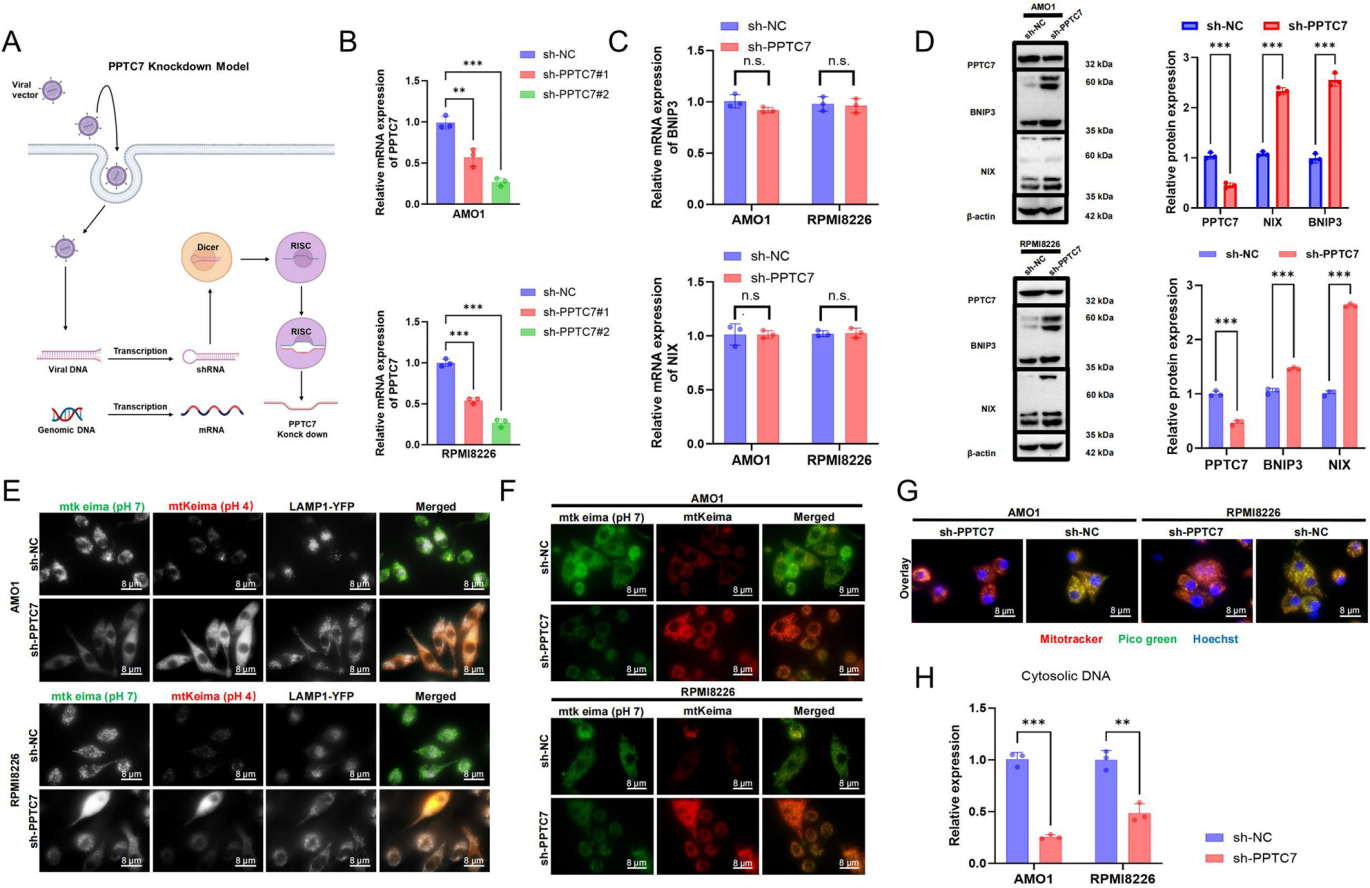
PPTC7 knockdown excessively activates mitophagy in MM cells.Note: **A** Schematic diagram of the experimental procedure illustrating lentiviral-mediated PPTC7 knockdown in AMO1 and RPMI8226 cells, created in BioRender; (**B**) RT-qPCR analysis of PPTC7 mRNA expression in AMO1 and RPMI8226 cells; (**C**) RT-qPCR detection of BNIP3 and NIX mRNA expression; (**D**) WB analysis of PPTC7, BNIP3, and NIX protein levels; (**E**) Confocal microscopy of mtKeima reporter co-localized with LAMP1-YFP, scale bar = 8 μm; (**F**) FACS and immunofluorescence analysis of mtKeima reporter to assess mitophagy activity, scale bar = 8 μm; (**G**-**H**) Immunofluorescence staining (scale bar = 8 μm) and RT-qPCR detection of cytosolic mtDNA release. All experiments were performed in triplicate. **p < 0.01, ***p < 0.001 indicate statistically significant differences between groups

Subsequently, PPTC7 was knocked down in AMO1 and RPMI8226 MM cells using lentiviral transduction (Fig. 3A). RT–qPCR showed that PPTC7 mRNA levels were markedly reduced in both sh-PPTC7#1 and sh-PPTC7#2 groups relative to sh-NC, confirming efficient knockdown in both cell lines. Because sh-PPTC7#2 produced the stronger silencing effect, it was used in subsequent experiments (Fig. 3B). Further assessment of BNIP3 and NIX mRNA levels showed no significant differences between the sh-PPTC7 and sh-NC groups in either AMO1 or RPMI8226 cells (Fig. 3C), indicating that PPTC7 knockdown did not affect the transcription of BNIP3 or NIX.

WB analysis of PPTC7, BNIP3, and NIX protein levels revealed that, relative to the sh-NC group, the expression of BNIP3 and NIX proteins was significantly upregulated in AMO1 and RPMI8226 cells in the sh-PPTC7 group, confirming that PPTC7 knockdown stabilized the protein levels of these mitophagy receptors (Fig. 3D). To monitor mitophagy activity, we utilized a mitochondria-targeted pH-sensitive reporter protein, mtKeima. Under neutral pH (cytoplasmic), mtKeima is excited at 440 nm (green channel), while under acidic pH (lysosomal), excitation occurs at 586 nm (red channel). In the sh-NC group, mtKeima signals co-localized modestly with the lysosomal marker LAMP1-YFP, indicating basal mitophagic flux. In contrast, this co-localization was markedly enhanced in the sh-PPTC7 group, suggesting significant activation of mitophagy (Fig. 3E). Further flow-cytometric and immunofluorescence analyses showed that, relative to the sh-NC group, sh-PPTC7 cells displayed stronger red fluorescence in the pH 4 channel and weaker green fluorescence. Merged images further revealed a marked increase in red puncta, consistent with enhanced mitochondrial delivery to lysosomes and increased mitophagy (Fig. 3F). Consistent with previous evidence that defective mitophagy promotes mitochondrial damage and cytoplasmic mtDNA release, we next examined mtDNA leakage after PPTC7 knockdown [29]. Immunofluorescence and RT–qPCR analyses showed that cytoplasmic mtDNA levels were significantly lower in sh-PPTC7 cells than in sh-NC cells, indicating that mitophagy activation reduced the release of mtDNA from damaged mitochondria into the cytoplasm (Fig. 3G-H).

These findings demonstrate that PPTC7 knockdown stabilizes BNIP3/NIX expression and leads to excessive activation of mitophagy in MM cells.

### PPTC7 inhibits mitophagy by regulating BNIP3 and NIX protein levels

**Fig. 4:**
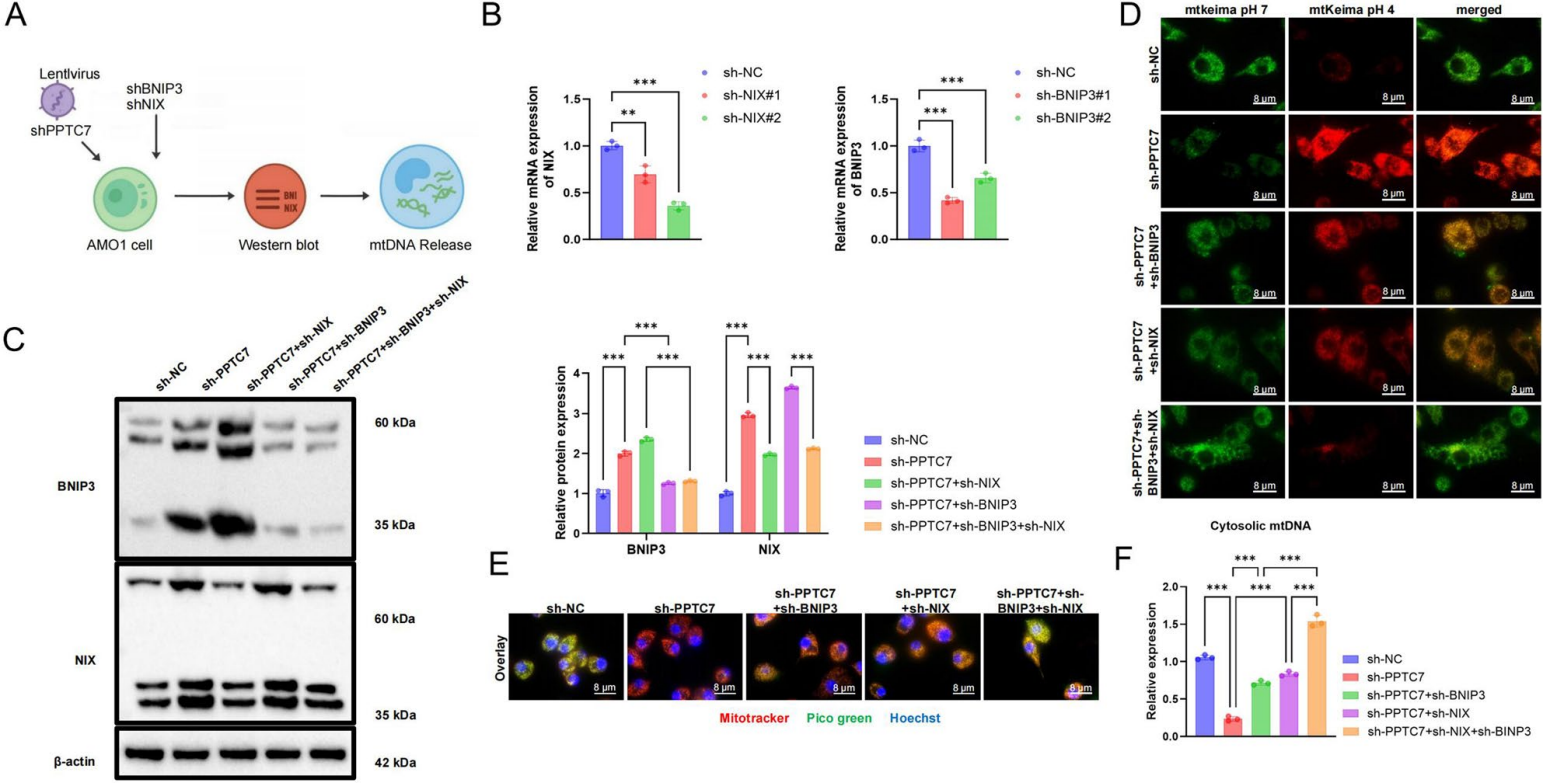
PPTC7 inhibits mitophagy by regulating the protein stability of BNIP3 and NIX.Note: **A** Schematic diagram of the experimental workflow for PPTC7, BNIP3, and NIX knockdown in AMO1 cells and subsequent analyses; (**B**) RT-qPCR analysis of BNIP3 and NIX mRNA levels in differently treated AMO1 cells; (**C**) WB analysis of PPTC7, BNIP3, and NIX protein expression in each group; (**D**) Mitophagy activity assessed using the mtKeima reporter in AMO1 cells across groups, analyzed by FACS and fluorescence microscopy, scale bar = 8 μm; (**E**-**F**) Immunofluorescence staining (scale bar = 8 μm) and RT-qPCR analysis of cytosolic mtDNA levels in AMO1 cells. All experiments were independently repeated three times. **p < 0.01, ***p < 0.001 indicate statistically significant differences between groups

Lentiviral transduction was used to knock down PPTC7, BNIP3, or NIX in AMO1 MM cells, generating the corresponding cell models (Fig. 4A). RT–qPCR confirmed efficient depletion of BNIP3 in the sh-BNIP3#1 and sh-BNIP3#2 groups and of NIX in the sh-NIX#1 and sh-NIX#2 groups relative to sh-NC. Because sh-BNIP3#1 and sh-NIX#2 produced the strongest knockdown, these constructs were used in subsequent experiments (Fig. 4B). WB analysis further showed that BNIP3 and NIX protein levels were markedly increased after PPTC7 silencing. This effect was reversed by co-silencing BNIP3 or NIX, and simultaneous knockdown of both genes substantially reduced the abundance of both proteins (Fig. 4C). Together, these results indicate that PPTC7 knockdown promotes the stabilization of BNIP3 and NIX proteins.

To further define the role of PPTC7 in mitophagy regulation, mitophagy activity was assessed in AMO1 cells using the mtKeima reporter system. Both flow cytometry and fluorescence microscopy showed that PPTC7 knockdown markedly increased mitophagy relative to the sh-NC group. This effect was attenuated by co-silencing BNIP3 or NIX and was further diminished when both genes were simultaneously knocked down (Fig. 4D). Cytosolic mtDNA release was then evaluated by immunofluorescence and RT–qPCR. Consistent with the increase in mitophagy, PPTC7 silencing significantly reduced cytosolic mtDNA levels, whereas additional knockdown of BNIP3, NIX or both restored mtDNA accumulation in the cytoplasm (Fig. 4E).

### PPTC7 promotes the ubiquitin-mediated degradation of BNIP3 and NIX in human MM cells

**Fig. 5:**
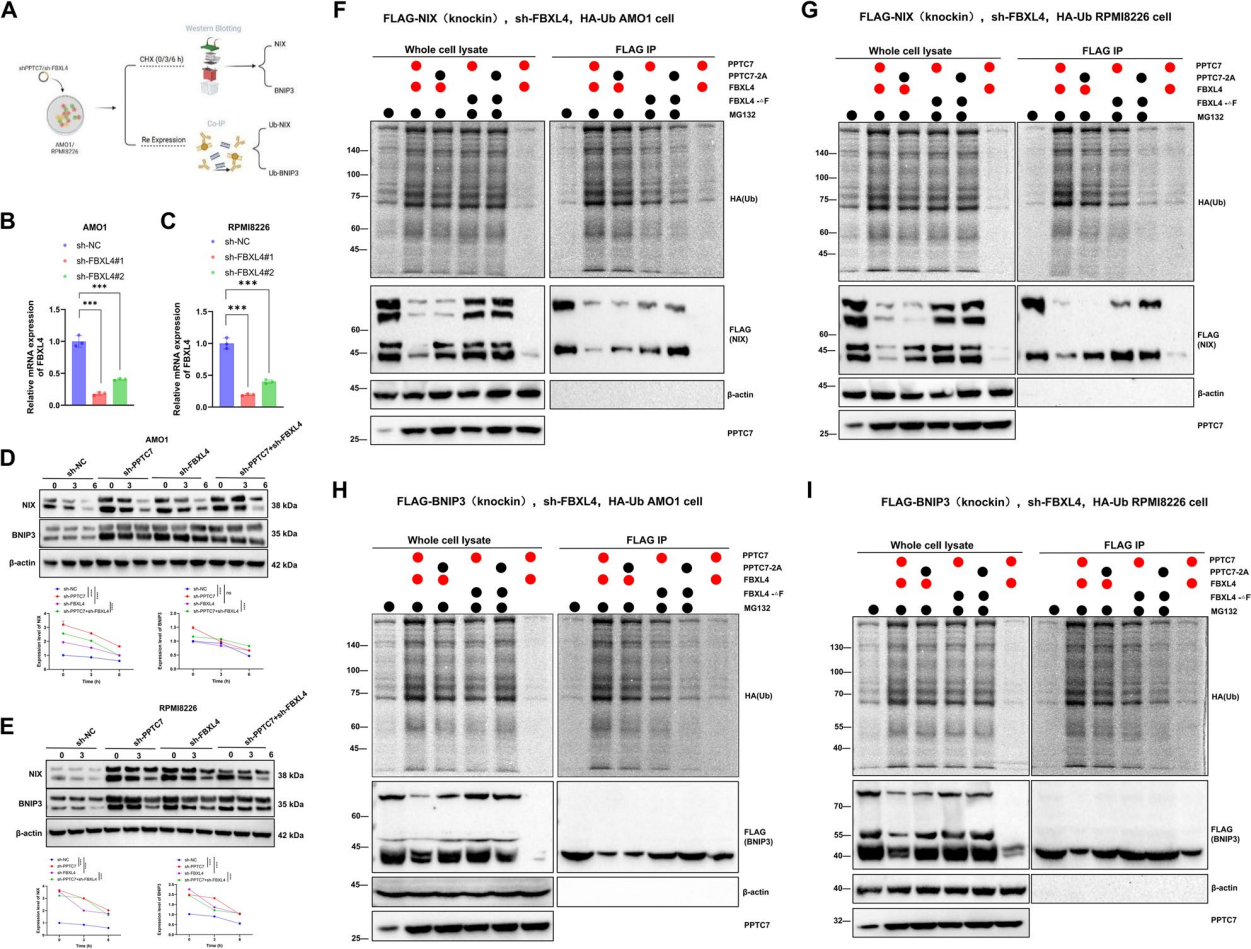
PPTC7 promotes SCFFBXL4-dependent ubiquitin degradation of BNIP3 and NIX in MM cells.Note: **A** Schematic illustration of the experimental workflow depicting PPTC7-mediated ubiquitin degradation of BNIP3 and NIX in AMO1 and RPMI8226 cells, created in BioRender; (**B**-**C**) RT-qPCR analysis of FBXL4 mRNA levels in AMO1 and RPMI8226 cells to confirm knockdown efficiency; (**D**-**E**) WB analysis of BNIP3 and NIX protein stability following CHX treatment in AMO1 and RPMI8226 cells; (**F**-**G**) Co-IP assays evaluating NIX ubiquitination levels and the regulatory effect of FBXL4 in AMO1 and RPMI8226 cells; (**H**-**I**) Co-IP analysis of BNIP3 ubiquitination to determine the role of PPTC7 in BNIP3 ubiquitin degradation. All experiments were performed in triplicate. ***p < 0.001, ****p < 0.0001 indicates statistically significant differences between groups

To determine whether PPTC7 regulates the FBXL4-dependent degradation of BNIP3 and NIX in MM cells, stable PPTC7- and FBXL4-silenced cell models were generated by lentiviral transduction (Fig. 5A). RT-qPCR analysis confirmed that, compared with the sh-NC group, FBXL4 mRNA expression was significantly reduced in both the sh-FBXL4#1 and sh-FBXL4#2 groups in AMO1 and RPMI8226 cells, with sh-FBXL4#1 showing a more pronounced knockdown efficiency and thus selected for subsequent experiments (Fig. 5B).

To determine the effect of PPTC7 on the ubiquitin-mediated degradation of BNIP3 and NIX, AMO1 and RPMI8226 cells were treated with cycloheximide (CHX) for 0, 3, or 6 h to assess protein stability. WB analysis revealed that BNIP3 and NIX protein levels were elevated and their half-lives were markedly prolonged in the sh-PPTC7 + CHX and sh-FBXL4 + CHX groups compared with the sh-NC + CHX group. However, the sh-PPTC7 + sh-FBXL4 + CHX group showed no further increase in BNIP3 and NIX protein levels relative to either single-knockdown group, suggesting that PPTC7 and FBXL4 act within the same pathway (Fig. 5D).

To further elucidate the mechanism by which PPTC7 regulates FBXL4-mediated ubiquitin degradation of BNIP3 and NIX, a ubiquitination detection system was established using FLAG-NIX and HA-Ub plasmids, or HA-Ub and FLAG-BNIP3 plasmids. Experiments were conducted in AMO1 and RPMI8226 cells with FBXL4 knockdown, and cells were treated with the proteasome inhibitor MG132. Co-IP analysis showed that ubiquitination of NIX was completely abolished in sh-FBXL4 cells (Fig. 5F-G, lane 1), while re-expression of FBXL4 restored NIX ubiquitination (Fig. 5F-G, lanes 2 and 3). Notably, co-expression of FBXL4 with PPTC7 further enhanced NIX ubiquitination (Fig. 5F-G, lane 2 vs. lane 3), whereas co-expression with an F-box domain-deficient mutant (FBXL4-ΔF) abrogated PPTC7-induced NIX ubiquitination (Fig. 5F-G, lane 4), indicating that PPTC7 promotes NIX ubiquitination via the SCF^FBXL4^ complex. A similar analysis of BNIP3 ubiquitination in AMO1 and RPMI8226 cells yielded consistent results (Fig. 5H-I).

Collectively, these findings identify PPTC7 as a critical rate-limiting factor in SCF^FBXL4^-dependent ubiquitination and degradation of BNIP3 and NIX.

### PPTC7 enhances mtDNA-dependent activation of the cGAS/STING pathway and promotes cellular senescence in MM cells by inhibiting mitophagy

**Fig. 6:**
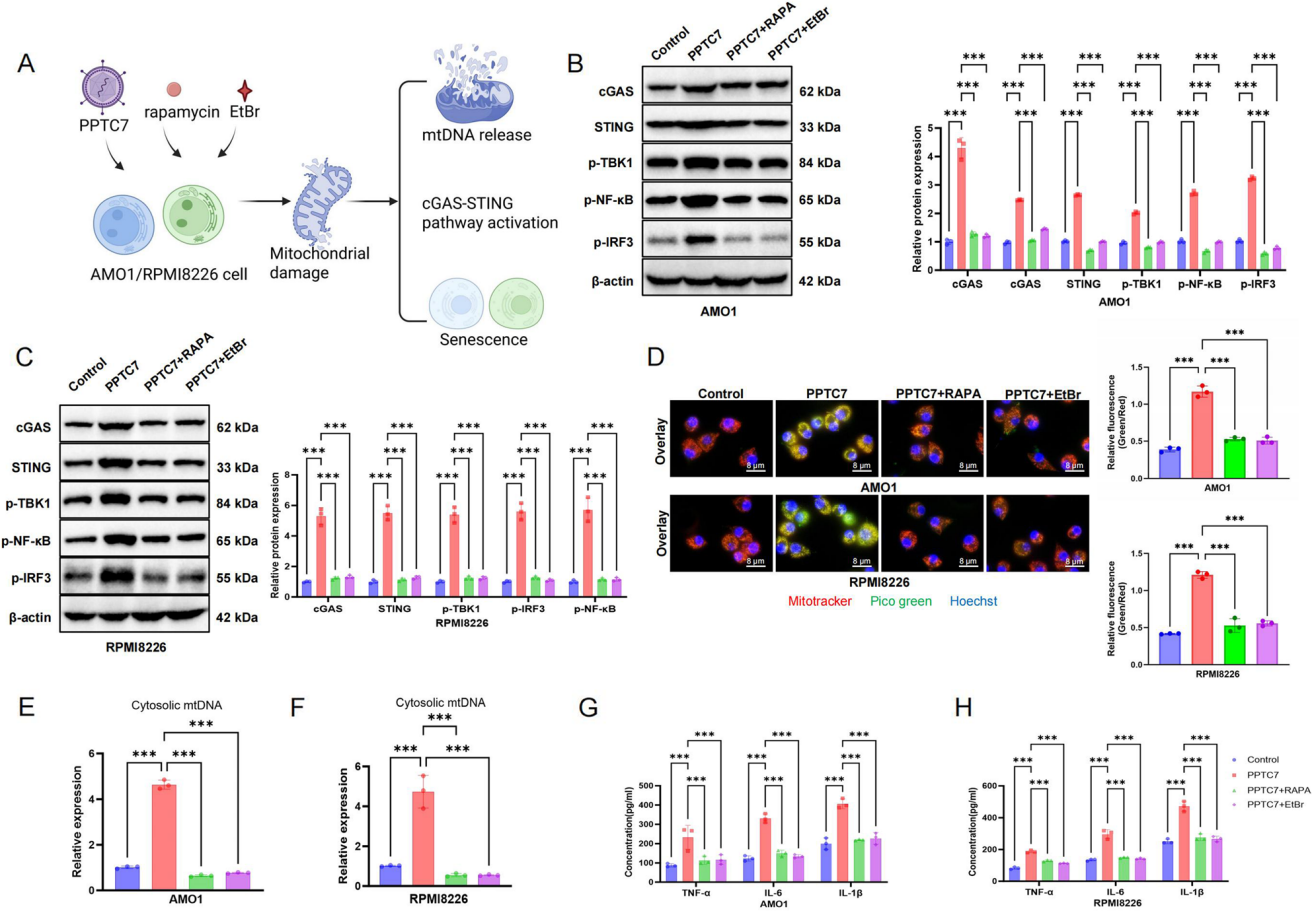
PPTC7 suppresses mitophagy and regulates mtDNA-dependent activation of the cGAS/STING pathway in MM cells.Note: **A** Schematic illustration of the experimental design in which PPTC7 was overexpressed via lentiviral transduction in AMO1 and RPMI8226 cells, followed by treatment with RAPA or EtBr, created in BioRender; (**B**-**C**) WB analysis of cGAS, STING, p-TBK1, p-IRF3, and p-NF-κB in AMO1 and RPMI8226 cells; (**D**-**F**) Immunofluorescence staining and RT-qPCR detection of cytosolic mtDNA release in the indicated groups (bar = 8 μm); (**G**-**H**) ELISA quantification of inflammatory cytokines TNF-α, IL-6, and IL-1β in the culture supernatants of AMO1 and RPMI8226 cells. All experiments were performed in triplicate. ***p < 0.001 indicates statistically significant differences between groups

To investigate the role of PPTC7 in regulating mtDNA-dependent activation of the cGAS/STING inflammatory pathway through modulation of mitophagy in MM cells, AMO1 and RPMI8226 cells were transduced with lentivirus to overexpress PPTC7 and subsequently treated with either RAPA or EtBr, the latter of which binds tightly to mtDNA and inhibits its replication during cell division. This enabled the establishment of various treatment groups (Fig. 6A). WB analysis showed that PPTC7 overexpression markedly increased the levels of cGAS, STING, p-TBK1, p-IRF3 and p-NF-κB relative to the control group. In contrast, these increases were attenuated by RAPA treatment, and a similar reduction was observed in the PPTC7 + EtBr group (Fig. 6B, C), supporting a role for mitophagy in regulating cGAS/STING pathway activation.

Cytosolic mtDNA release was then assessed. Immunofluorescence and RT–qPCR analyses showed that PPTC7 overexpression significantly increased cytoplasmic mtDNA levels in both AMO1 and RPMI8226 cells relative to control cells. mtDNA accumulation was markedly reduced after treatment with RAPA or EtBr, indicating that restoration of mitophagy or depletion of mtDNA attenuated mtDNA leakage induced by PPTC7 (Fig. 6D–F). ELISA further showed that TNF-α, IL-6 and IL-1β levels were elevated in the PPTC7 group but were substantially reduced in the PPTC7 + RAPA and PPTC7 + EtBr groups (Fig. 6G).

To determine whether PPTC7 promotes MM-cell senescence through cGAS/STING signaling, cGAS was silenced in MM cells. RT–qPCR confirmed effective knockdown in both sh-cGAS#1 and sh-cGAS#2 groups, with sh-cGAS#2 showing greater silencing efficiency and therefore being used in subsequent experiments (Figure S6A-B). Furthermore, WB analysis showed that PPTC7 overexpression increased the levels of cGAS, STING, p-TBK1, p-IRF3 and p-NF-κB relative to the sh-NC+vector group in both AMO1 and RPMI8226 cells, whereas these increases were markedly attenuated after cGAS knockdown (Figure S6C-D).

Markers of cellular senescence were assessed across different treatment groups. SA-β-gal staining showed that the proportion of senescent cells in both AMO1 and RPMI8226 cells was significantly increased in the PPTC7 + sh-NC group relative to the sh-NC+vector group, whereas cGAS knockdown markedly reduced the fraction of senescent cells in the PPTC7 background (Figure S6E-F). WB analysis further demonstrated that p16 and p21 levels were elevated in the PPTC7 + sh-NC group but were substantially decreased in the PPTC7 + sh-cGAS group (Figure S6G-H).

These findings indicate that PPTC7 promotes MM-cell senescence by activating the mtDNA-dependent cGAS/STING inflammatory pathway through mitophagy regulation.

### The PPTC7/BNIP3/NIX axis inhibits mitophagy to activate the cGAS/STING signaling pathway and enhance sensitivity of MM cells to CAR-T therapy

To determine whether PPTC7-mediated mitophagy regulates MM-cell sensitivity to CAR-T therapy, AMO1 and RPMI8226 cells were engineered to overexpress PPTC7 and were treated with RAPA. WB analysis showed that PPTC7 overexpression reduced the LC3-II/LC3-I ratio and increased p62 accumulation relative to the control group, consistent with suppression of mitophagy. These effects were reversed by RAPA treatment, which increased the LC3-II/LC3-I ratio and reduced p62 levels in PPTC7-overexpressing cells (Figure S7A-B). Immunofluorescence and flow-cytometric analyses further showed that ΔΨm was decreased in the PPTC7 group but was restored after RAPA treatment (Figure S7C-D).

The treated MM cells were then co-cultured with BCMA CAR-T cells, and granzyme B expression in CAR-T cells was measured by flow cytometry. Granzyme B levels were significantly increased in the PPTC7 group relative to the control group, whereas this increase was attenuated in the PPTC7 + RAPA group (Figure S7E-F). Consistently, CAR-T cytotoxicity assays showed that apoptosis of CFSE-labeled target cells was enhanced by PPTC7 overexpression but reduced after RAPA treatment (Figure S7G). These findings suggest that PPTC7 enhances the sensitivity of MM cells to CAR-T therapy by inhibiting mitophagy.

**Fig. 7:**
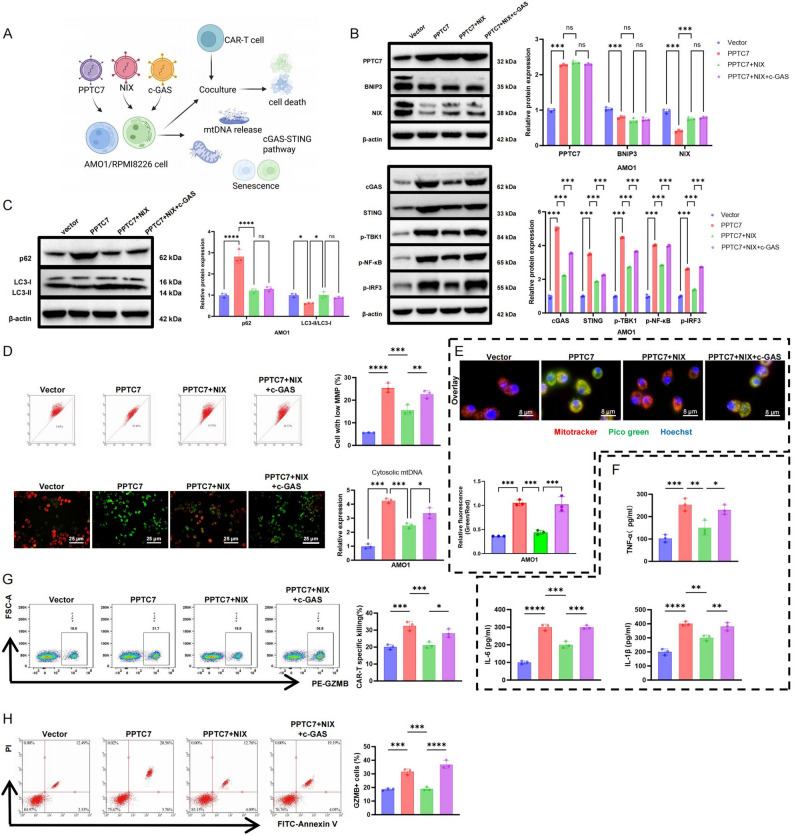
PPTC7 regulates AMO1 cell sensitivity to CAR-T therapy by activating the cGAS/STING signaling pathway through modulation of the BNIP3/NIX axis.Note: **A** Schematic diagram illustrating the construction of AMO1 cell models overexpressing PPTC7, NIX, and cGAS, created in BioRender; (**B**) WB analysis of PPTC7, BNIP3, NIX, and key proteins in the cGAS/STING pathway across different treatment groups; (**C**) WB detection of LC3-II/LC3-I ratios and p62 accumulation levels; (**D**) JC-1 staining and flow cytometry analysis of ΔΨm (bar = 25 μm; red and green fluorescence represent JC-1 aggregates and monomers, respectively); (**E**) Immunofluorescence staining and RT-qPCR assessment of cytosolic mtDNA levels (bar = 8 μm); (**F**) ELISA quantification of inflammatory cytokines TNF-α, IL-6, and IL-1β in different groups; (**G**) Flow cytometric analysis of granzyme B expression in CAR-T; (**H**) Apoptosis rate of CFSE-labeled AMO1 cells evaluated by PI staining. All experiments were conducted in triplicate. *p < 0.05, **p < 0.01, ***p < 0.001, ****p < 0.0001 indicate statistical significance between groups

To determine whether PPTC7 enhances MM-cell sensitivity to CAR-T therapy through the BNIP3/NIX axis, mitophagy suppression and cGAS/STING activation, AMO1 and RPMI8226 cells overexpressing PPTC7, NIX and cGAS were generated (Fig. 7A). WB analysis showed that PPTC7 overexpression increased PPTC7 abundance and activated the cGAS/STING pathway, while reducing BNIP3 and NIX levels relative to the vector group. Restoration of NIX expression in PPTC7-overexpressing cells reduced cGAS/STING pathway activation without altering PPTC7 or BNIP3 levels. Re-expression of cGAS in the PPTC7 + NIX background reactivated cGAS/STING signaling, again without affecting PPTC7, BNIP3 or NIX expression (Fig. 7B and Figure S8A).

Mitophagy activity was then evaluated by measuring the LC3-II/LC3-I ratio and p62 accumulation. Compared with vector controls, PPTC7 overexpression decreased the LC3-II/LC3-I ratio and increased p62 levels, consistent with mitophagy inhibition. These changes were reversed by NIX overexpression, which restored the LC3-II/LC3-I ratio and reduced p62 accumulation. By contrast, cGAS re-expression in PPTC7 + NIX cells did not alter either LC3 processing or p62 levels (Fig. 7 C and Figure S8B).

ΔΨm and cytoplasmic mtDNA levels were next examined. Relative to vector controls, PPTC7-overexpressing cells showed a marked reduction in ΔΨm and an increase in cytoplasmic mtDNA. These effects were partially reversed by NIX overexpression, which restored ΔΨm and reduced cytoplasmic mtDNA accumulation. Re-expression of cGAS in PPTC7 + NIX cells again reduced ΔΨm and further enhanced mtDNA-associated stress responses (Fig. 7D and Figure S8C-D). Consistently, ELISA showed that inflammatory cytokine levels were elevated in the PPTC7 group, suppressed in the PPTC7 + NIX group and increased again after cGAS re-expression (Fig. 7 F and Figure S8E).

BCMA CAR-T cells were then co-cultured with the indicated MM cells, and granzyme B expression was measured by flow cytometry. Granzyme B levels were higher in the PPTC7 group than in the vector group, decreased in the PPTC7 + NIX group and rose again in the PPTC7 + NIX+cGAS group (Fig. 7G and Figure S8F). Similarly, CAR-T cytotoxicity assays showed that target-cell apoptosis was increased by PPTC7 overexpression, reduced by NIX restoration and re-elevated after cGAS re-expression (Fig. 7H and Figure S8G).

These results indicate that PPTC7 enhances the sensitivity of MM cells to CAR-T therapy by downregulating BNIP3/NIX expression, thereby inhibiting mitophagy and activating the cGAS/STING signaling pathway.

### In vivo animal experiments confirm That the PPTC7/BNIP3/NIX Axis suppresses tumor growth in a MM mouse model

In vivo, a NOD SCID xenograft model was used to examine whether PPTC7 regulates MM progression through the BNIP3/NIX axis, mitophagy inhibition and cGAS/STING activation (Figure S9A). Tumor weight and volume were both significantly reduced in the PPTC7 group relative to the vector group, whereas NIX restoration markedly increased both parameters compared with PPTC7 overexpression alone (Figure S9B-D). IHC analysis further showed that PPTC7 expression was elevated and BNIP3/NIX expression was reduced in tumors from the PPTC7 group. Re-expression of NIX in the PPTC7 background restored NIX levels without affecting PPTC7 or BNIP3 expression (Figure S9E).

WB analysis of xenograft tumors showed that PPTC7 overexpression increased the abundance of cGAS/STING pathway proteins and the senescence markers p16 and p21 relative to the vector group. This was accompanied by a reduced LC3-II/LC3-I ratio and increased p62 accumulation, consistent with mitophagy suppression. These changes were reversed by NIX re-expression, which attenuated cGAS/STING activation and senescence-marker expression while restoring LC3 processing and reducing p62 levels (Figure S9F-H). Furthermore, TEM of tumor tissues demonstrated that mitophagy was significantly suppressed in the PPTC7 group relative to the vector group, whereas mitophagy was reactivated in the PPTC7 + NIX group compared with the PPTC7 group (Figure S9I). ELISA results further indicated that inflammatory cytokine levels were significantly elevated in the PPTC7 group compared with the vector group, and significantly decreased in the PPTC7 + NIX group relative to the PPTC7 group (Figure S9J).

These findings suggest that PPTC7 inhibits tumor growth in MM by modulating mitophagy and activating the cGAS/STING signaling pathway.

### In vivo animal experiments validate that PPTC7 inhibits mitophagy and enhances CAR-T-mediated cytotoxicity

**Fig. 8:**
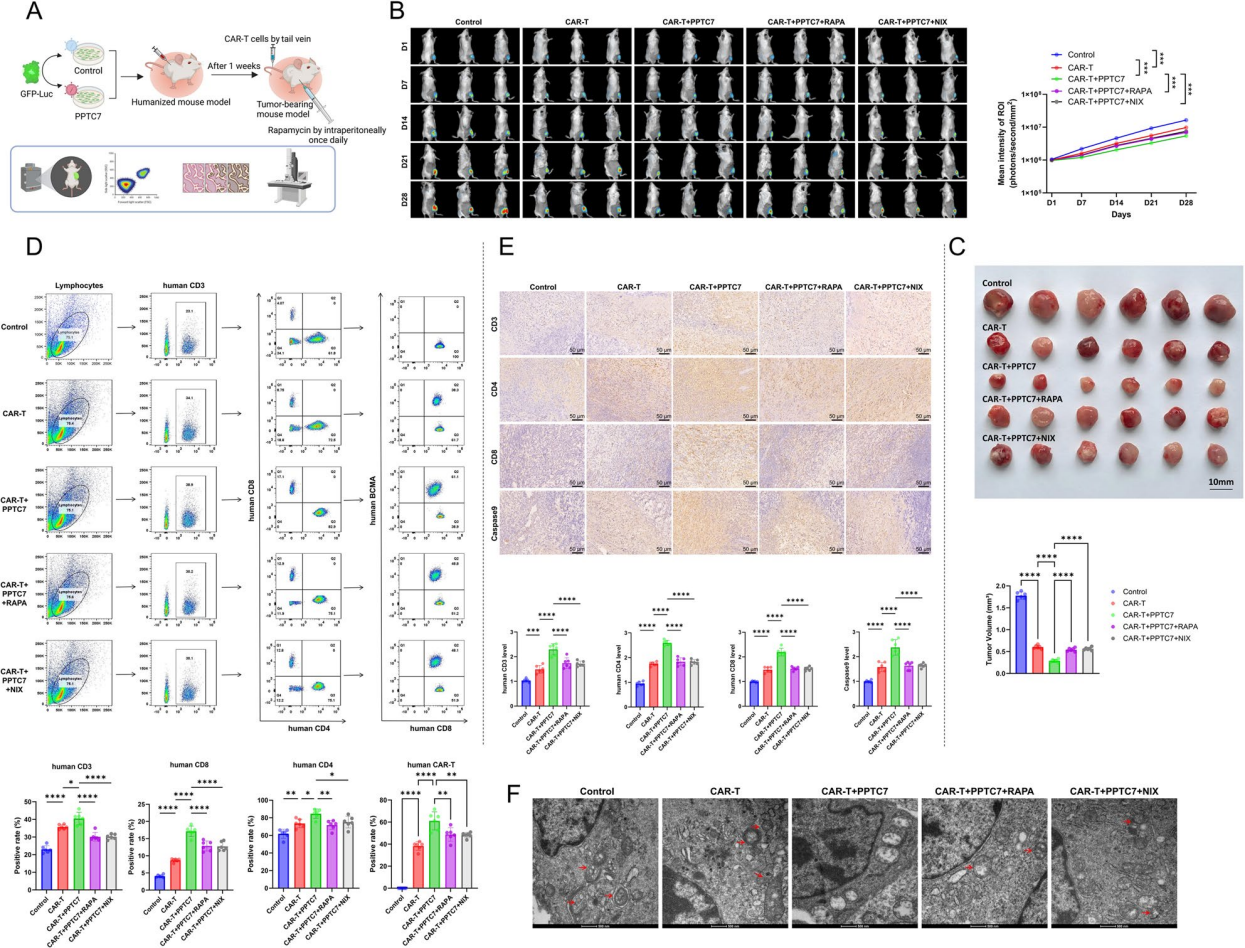
PPTC7-mediated regulation of mitophagy influences CAR-T-mediated tumor killing.Note: **A** Schematic illustration of the experimental workflow using NOD SCID immunodeficient mice to evaluate the effect of PPTC7-mediated inhibition of mitophagy on CAR-T therapy, created in BioRender; (**B**) BLI analysis of tumor burden in each group of mice, where BLI signal intensity reflects tumor load; (**C**) Representative images and statistical analysis of dissected tumors and tumor weights in each group; (**D**) Flow cytometry analysis of tumor-infiltrating CD3⁺, CD4⁺, and CD8⁺ T cells, as well as activated CAR-T cells in xenograft tissues; (**E**) IHC staining of CD3, CD4, CD8, and the apoptosis-related protein Caspase-9 in tumor tissues (bar = 50 μm); (**F**) TEM visualization of mitophagy in xenograft tissues (bar = 500 nm). Six mice were used per group. **p < 0.01, ***p < 0.001, ****p < 0.0001 indicate statistical significance between groups

To further investigate the role of PPTC7-mediated inhibition of mitophagy in CAR-T therapy, a NOD SCID immunodeficient mouse model was employed (Fig. 8A). Tumor burden was monitored longitudinally by bioluminescence imaging. Relative to the control group, CAR-T treatment significantly reduced tumor burden. This antitumor effect was further enhanced in the CAR-T+PPTC7 group, whereas co-treatment with RAPA or NIX restoration partially reversed the PPTC7-mediated reduction in tumor burden (Fig. 8B). Tumor weight measurements further confirmed these findings (Fig. 8C).

Flow-cytometric analysis of xenograft tumors showed that CAR-T treatment significantly increased the infiltration of CD3^+^, CD4^+^ and CD8^+^ T cells, as well as activated CAR-T cells, relative to the control group. This increase was further enhanced in the CAR-T+PPTC7 group. By contrast, T-cell and activated CAR-T-cell infiltration was reduced in the CAR-T+PPTC7 + RAPA and CAR-T+PPTC7 + NIX groups compared with the CAR-T+PPTC7 group, indicating that mitophagy inhibition facilitates immune-cell accumulation within the tumor microenvironment (Fig. 8D).

IHC staining analysis revealed that CD3, CD4, and CD8 cell expression levels were significantly elevated in the tumor tissues of CAR-T group mice compared with the Control group, accompanied by a marked increase in Caspase 9 expression. Compared with the CAR-T group, the CAR-T+PPTC7 group exhibited further enhanced expression of CD3, CD4, and CD8 cells, along with a significant upregulation of Caspase 9. In contrast, the CAR-T+PPTC7 + RAPA and CAR-T+PPTC7 + NIX groups showed reduced levels of CD3, CD4, and CD8 cell expression, as well as decreased Caspase 9 expression, relative to the CAR-T+PPTC7 group, suggesting that inhibition of mitophagy may enhance immune responses and promote tumor cell apoptosis (Fig. 8E).

TEM was employed to observe mitochondrial ultrastructural changes in xenograft tumor tissues. No significant alterations in mitophagy were observed in the CAR-T group compared with the Control group. However, mitophagy was markedly suppressed in the CAR-T+PPTC7 group and was restored by RAPA treatment or NIX re-expression (Fig. 8F).

These findings suggest that PPTC7 enhances CAR-T-mediated tumor cytotoxicity by inhibiting mitophagy through the BNIP3/NIX axis and stimulating the cGAS/STING signaling pathway.

## Discussion

CAR-T therapy has become an important treatment option for relapsed or refractory MM, with BCMA-targeted CAR-T products demonstrating remarkable clinical efficacy [30, 31]. However, a proportion of patients relapse rapidly or acquire resistance soon after initial treatment, which substantially compromises the durability of CAR-T therapy [32]. Most existing studies have focused on “extrinsic” mechanisms related to the CAR-T themselves, including T cell exhaustion, upregulation of immune checkpoints, and tumor antigen loss [33]. In contrast, the role of “intrinsic” adaptive mechanisms within tumor cells in shaping therapeutic response remains insufficiently explored [34]. The present study addresses this gap by investigating tumor cell-intrinsic metabolic and autophagic regulatory mechanisms, revealing how mitophagy modulates CAR-T efficacy and offering novel insights into overcoming treatment resistance.

Mitophagy is a critical cellular process that maintains mitochondrial homeostasis under stress conditions [35, 36]. Recent studies suggest that tumor cells can enhance mitophagy to eliminate damaged mitochondria, thereby preventing the release of mtDNA and suppressing the activation of innate immune pathways, ultimately facilitating immune evasion [11, 37]. In the setting of immunotherapy, autophagy-dependent suppression of cGAS/STING signaling may further weaken antitumor immune responses [9]. Functional experiments in this study demonstrate that inhibition of mitophagy promotes mtDNA release and activates the cGAS/STING pathway, thereby sensitizing MM cells to CAR-T-mediated cytotoxicity. These findings identify mitophagy as a key regulator of immune tolerance and CAR-T resistance in MM.

BNIP3 and NIX are key mitophagy adaptors, traditionally recognized for being upregulated under stress conditions to facilitate the identification and degradation of damaged mitochondria [13]. This study found that PPTC7 primarily suppresses mitophagy by regulating the ubiquitin-dependent degradation of BNIP3 and NIX, thereby reducing their stability. Concurrently, we observed moderate alterations in general autophagic flux, indicating possible crosstalk between the pathways; however, this did not alter the predominant role of mitophagy in our study. Previous research has shown that BNIP3 and NIX mainly govern the mitophagy process, but their knockdown can also affect general autophagy, leading to decreased LC3II/LC3I ratios and increased p62 expression. Our findings are consistent with these reports [38]. This finding challenges the conventional view of BNIP3 and NIX solely as “autophagy activators” and suggests that their function in MM may be constrained by specific mechanisms governing protein homeostasis. Elucidating this regulatory pathway not only deepens our understanding of the mitophagy network but also provides a novel theoretical framework for immunoenhancement strategies targeting the BNIP3/NIX axis.

Existing studies have shown that PPTC7 is a mitochondria-localized phosphatase of the PP2C family that plays a critical role in maintaining mitochondrial quality control. Its precursor can be sequestered on the outer mitochondrial membrane by BNIP3/NIX and assembled into a “substrate-PPTC7-SCF^FBXL4^” complex, which facilitates the ubiquitin-dependent degradation of BNIP3/NIX and thereby suppresses BNIP3/NIX-dependent mitophagy. In addition, PPTC7 possesses a weak mitochondrial targeting sequence (MTS) and is upregulated under stress conditions such as starvation, features that collectively enable its fine-tuned regulation of receptor-mediated mitophagy [13, 39]. However, current research on PPTC7 in tumors remains limited. In certain solid tumor models, such as cervical cancer, BNIP3/NIX-mediated mitophagy has been shown to participate in hypoxia adaptation, metabolic reprogramming, and treatment resistance [38, 40]. PPTC7 is frequently implicated as an upstream suppressor in this axis and has been suggested to influence mitochondrial homeostasis and cell survival. Nevertheless, these studies have primarily focused on basic metabolism or non-tumor models, and investigations into the role of the PPTC7-BNIP3/NIX axis in cancer—particularly in hematologic malignancies—are scarce. To date, no direct evidence has linked PPTC7 with CAR-T therapy, representing a key gap that this study aims to address.

In this study, we demonstrated that upregulation of PPTC7 in MM suppressed tumor-derived mitophagy, promoted mtDNA release, and activated the cGAS/STING pathway. This activation not only induced senescence-associated phenotypes in myeloma cells but also enhanced the secretion of inflammatory cytokines, thereby augmenting the cytotoxic activity of CAR-T cells. Thus, PPTC7 sensitized CAR-T therapy through a sequential mechanism involving “PPTC7-mitophagy inhibition-mtDNA release-cGAS/STING activation-senescence/immune potentiation.” From this perspective, PPTC7 serves as an upstream regulator of the BNIP3/NIX-selective autophagy pathway and links mitochondrial quality control with innate immunity via the mtDNA-cGAS/STING axis. This offers a tumor-intrinsic immunopotentiation strategy distinct from exogenous inflammatory stimulation. Our findings not only provide new evidence for the role of PPTC7 in hematologic malignancies but also extend the mechanistic understanding of the PPTC7-BNIP3/NIX axis in the context of immunotherapy. Compared with previous studies, the novelty of our work lies in the integration of mitophagy regulation with CAR-T sensitivity, thereby identifying a promising therapeutic target.

The cGAS/STING signaling axis is a central innate immune pathway that senses cytosolic DNA and drives type I interferon responses [41]. Previous studies have suggested its capacity to enhance T cell responses and promote antitumor effects in the context of radiotherapy, chemotherapy, and immunotherapy [42]. In this study, we introduced this pathway into the setting of CAR-T therapy and demonstrated for the first time that PPTC7-mediated inhibition of mitophagy leads to mtDNA leakage, which activates the cGAS/STING pathway, induces cellular senescence in MM cells, and ultimately enhances CAR-T cytotoxicity. These findings suggest that modulation of cGAS/STING activity may serve as a promising strategy to potentiate CAR-T efficacy and offer a mechanistic rationale for the combined use of immunostimulatory agents.

To ensure the scientific rigor and reliability of our findings, we employed an integrated approach combining multi-omics analysis, bioinformatic prediction, cellular functional assays, and in vivo mouse models. Starting from database screening of the PPTC7/BNIP3L axis, we sequentially conducted autophagy assessments, CAR-T co-culture experiments, and analyses of tumor burden and T cell infiltration at the animal level, thereby reinforcing the central hypothesis of the study. Furthermore, reversal of the CAR-T enhancement by autophagy inducers confirmed the specificity of the proposed mechanism. This research framework, which bridges database mining with preclinical validation, substantially strengthens the translational potential of our results.

Most current studies on CAR-T resistance focus on T cell exhaustion, antigen loss, or immunosuppressive factors within the tumor microenvironment, while investigations into how tumor cells intrinsically regulate therapeutic responses remain relatively underexplored. In this study, we examined tumor-derived mitophagy and revealed that tumor cells actively modulate metabolic and inflammatory pathways to influence CAR-T cytotoxicity, thereby expanding the mechanistic landscape of CAR-T immune evasion. Notably, this is the first report to establish a functional linkage among PPTC7, BNIP3/NIX, and the cGAS/STING pathway, offering a novel theoretical framework for understanding the intersection of immunotherapy and cellular metabolic regulation. This distinct mechanistic insight highlights the study’s unique contribution in terms of target innovation and mechanistic depth.

We systematically demonstrate that PPTC7 suppresses mitophagy by promoting the ubiquitin-mediated degradation of BNIP3/NIX, thereby activating the mtDNA-dependent cGAS/STING pathway and enhancing CAR-T-mediated cytotoxicity against MM. This newly identified molecular mechanism offers a novel explanation for CAR-T resistance and positions PPTC7 as a potential immunosensitization target with clinical translational relevance. Scientifically, this study establishes a functional connection among autophagy, inflammation, and therapeutic response in tumor cells, presenting significant theoretical implications. Clinically, targeting PPTC7 or its downstream pathways may enhance the efficacy of CAR-T therapy and prolong remission duration. However, limitations remain: the expression characteristics of this pathway in clinical specimens were not systematically evaluated, and pharmacological modulators of PPTC7 are still underdeveloped. In the future, the universality and tissue specificity of the PPTC7–BNIP3/NIX axis should be systematically evaluated across other tumor types, including hematologic malignancies. It is also important to elucidate its interactions with HIF-1α/hypoxia, the functional status of FBXL4, and metabolic pathways. In addition, the potential for rational combination therapies involving STING agonists, general or mitophagy-specific autophagy modulators, and CAR-T or immune checkpoint inhibitors warrants thorough investigation to validate its translational potential in tumor immunotherapy. Moreover, efforts should be directed toward the development of PPTC7-targeted agents and the assessment of their applicability across various immunotherapeutic platforms, with the aim of advancing PPTC7 as a broad-spectrum immune-enhancing strategy.

## Conclusion

This study systematically defines a central mechanism by which the mitophagy regulator PPTC7 controls the sensitivity of MM cells to CAR-T therapy. PPTC7 promoted the ubiquitin-mediated degradation of BNIP3 and NIX, thereby suppressing tumor-cell mitophagy. Mitophagy inhibition led to cytosolic accumulation of mtDNA, activation of cGAS/STING signaling, induction of cellular senescence and enhancement of CAR-T-mediated cytotoxicity. Both in vitro and in vivo findings showed that PPTC7 expression improved the antitumor efficacy of CAR-T therapy, supporting its role in overcoming immunotherapy resistance. These results further suggest that MM cells may evade CAR-T killing by restraining mitophagy-dependent immune activation and identify the PPTC7/BNIP3/NIX axis as a potential therapeutic target. Targeting this pathway, alone or in combination with autophagy-modulating strategies, may provide a promising approach for improving CAR-T efficacy in MM.

## Supplementary Information

Supplementary Material 1.

Supplementary Material 2.

Supplementary Material 3.

Supplementary Material 4.

Supplementary Material 5.

Supplementary Material 6.

Supplementary Material 7.

Supplementary Material 8.

Supplementary Material 9.

Supplementary Material 10.

## Data Availability

All data generated or analyzed during this study are included in this article and/or its supplementary material files. Further inquiries can be directed to the corresponding author.

## Abbreviations

ANOVA: Analysis of Variance
BCMA: B-Cell Maturation Antigen
BLI: Bioluminescence Imaging
BP: Biological Process
CAR-T: Chimeric Antigen Receptor T Cell
CC: Cellular Component
CHX: Cycloheximide
Co-IP: Co-Immunoprecipitation
DEGs: Differentially Expressed Genes
E: T: Effector-to-Target
ELISA: Enzyme-Linked Immunosorbent Assay
EtBr: Ethidium Bromide
FBS: Fetal Bovine Serum
FBXL4: F-Box And Leucine Rich Repeat Protein 4
GEO: Gene Expression Omnibus
GO: Gene Ontology
HA-Ub: HA-Tagged Ubiquitin
H_2_O_2_: Hydrogen Peroxide
IHC: Immunohistochemical
KEGG: Kyoto Encyclopedia of Genes and Genomes
MF: Molecular Function
MM: Multiple Myeloma
mtDNA: Mitochondrial DNA
nDNA: Nuclear DNA
PCA: Principal Component Analysis
PBS: Phosphate-Buffered Saline
PBMCs: Peripheral Blood Mononuclear Cells
PCs: Principal Components
PI: Propidium Iodide
PPI: Protein-Protein Interaction
PPTC7: Protein Phosphatase Targeting Cofactor 7
RAPA: Rapamycin
RT-qPCR: Reverse Transcription Quantitative Polymerase Chain Reaction
scRNA-seq: Single-Cell RNA Sequencing
SD: Standard Deviation
TEM: Transmission Electron Microscopy
WB: Western Blot
ΔΨm: Mitochondrial Membrane Potential
