# Supplementary figures and images for: The PPTC7/BNIP3/NIX axis induces cGAS/STING-mediated senescence and augments CAR-T efficacy by repressing tumor-intrinsic mitophagy
Source: J Exp Clin Cancer Res. 2026 Apr 18;45:135. doi: 10.1186/s13046-026-03713-7 (PMC13270736; doi:10.1186/s13046-026-03713-7)

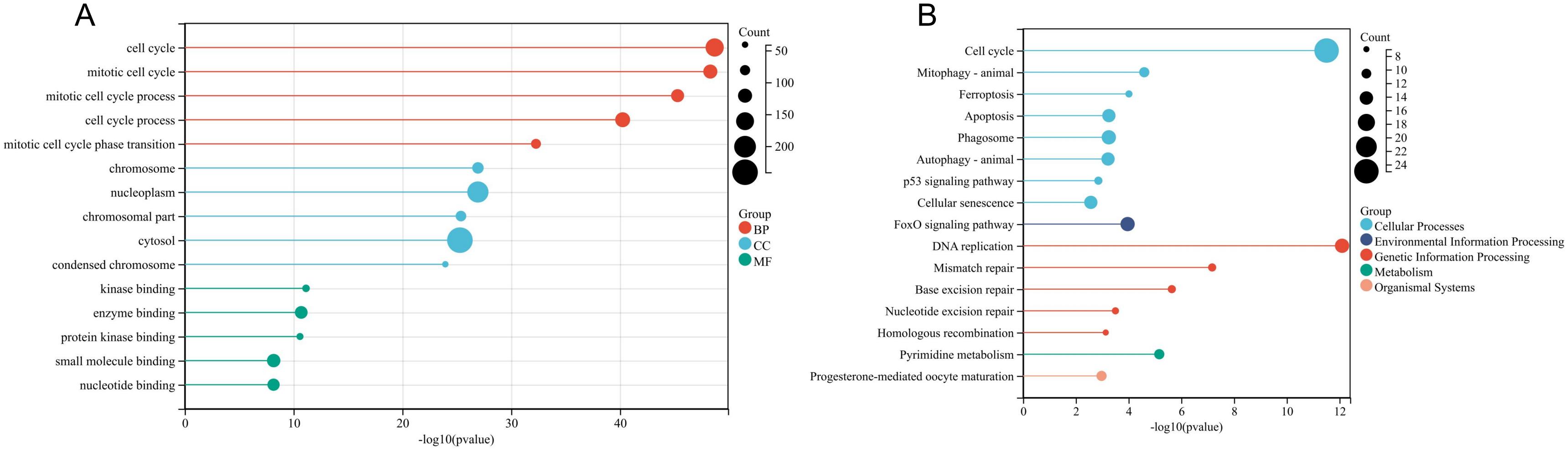

Supplement: Supplementary file 1 — Supplementary Material 1. [file 13046_2026_3713_MOESM1_ESM.jpg]

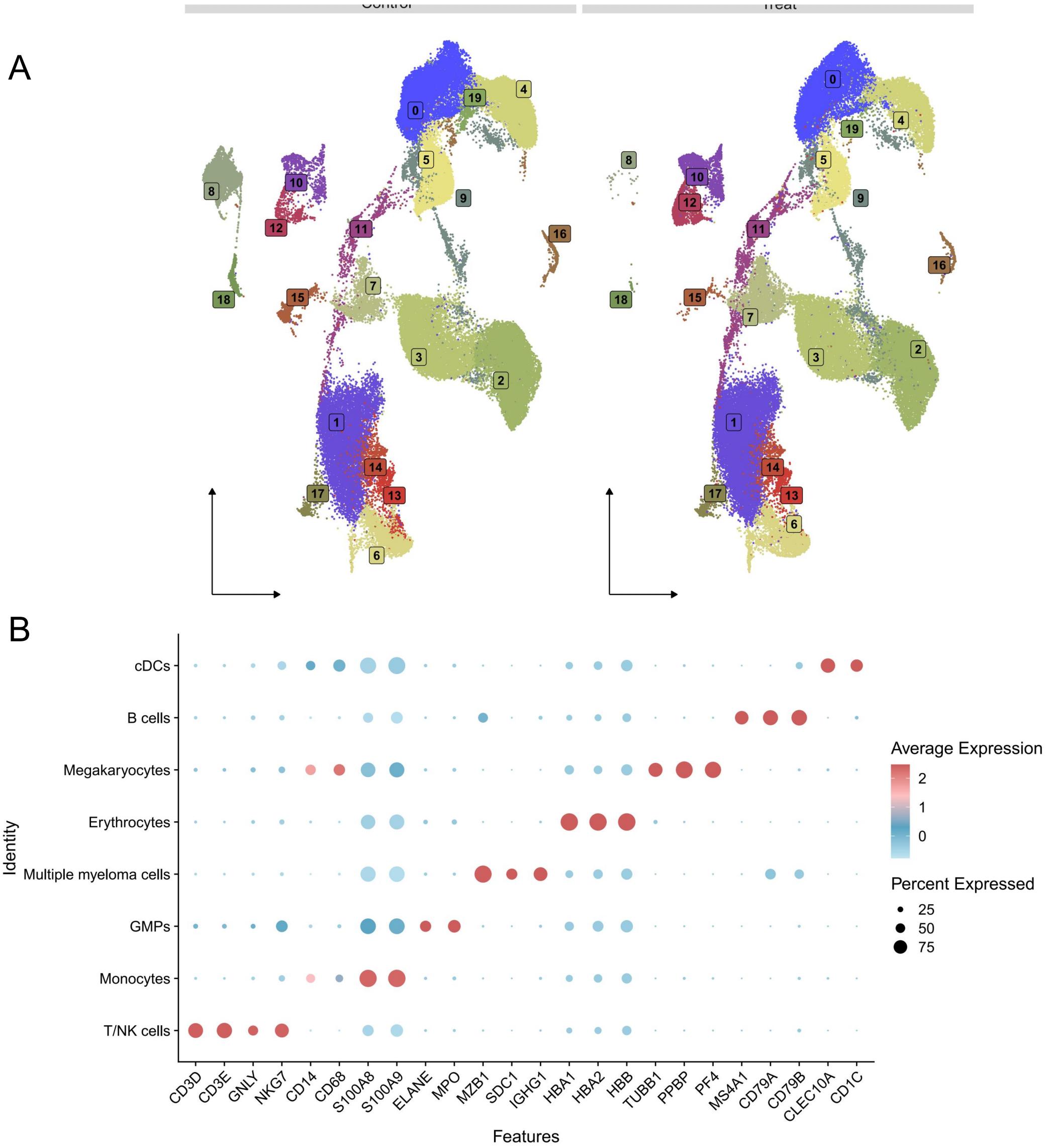

Supplement: Supplementary file 2 — Supplementary Material 2. [file 13046_2026_3713_MOESM2_ESM.jpg]

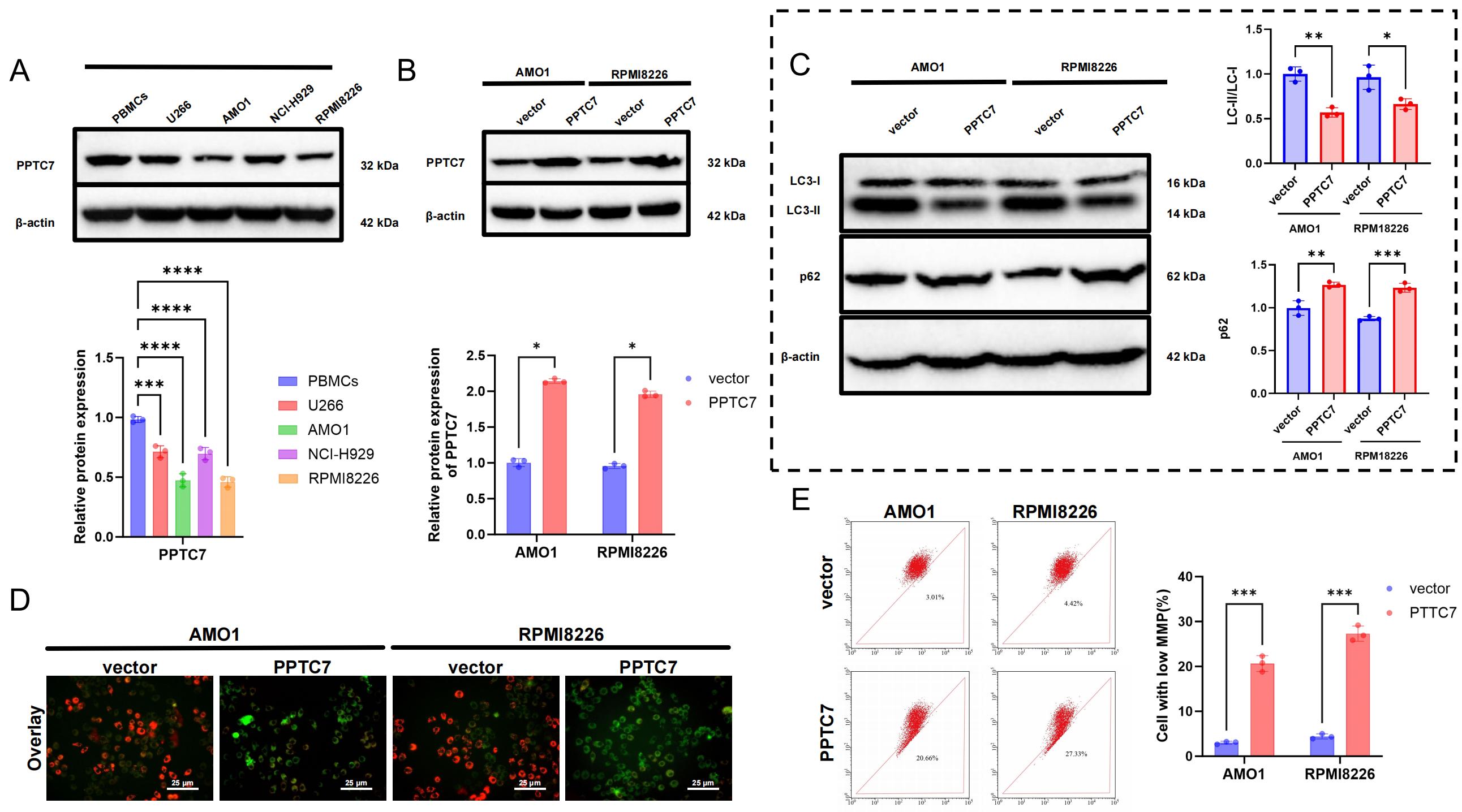

Supplement: Supplementary file 3 — Supplementary Material 3. [file 13046_2026_3713_MOESM3_ESM.jpg]

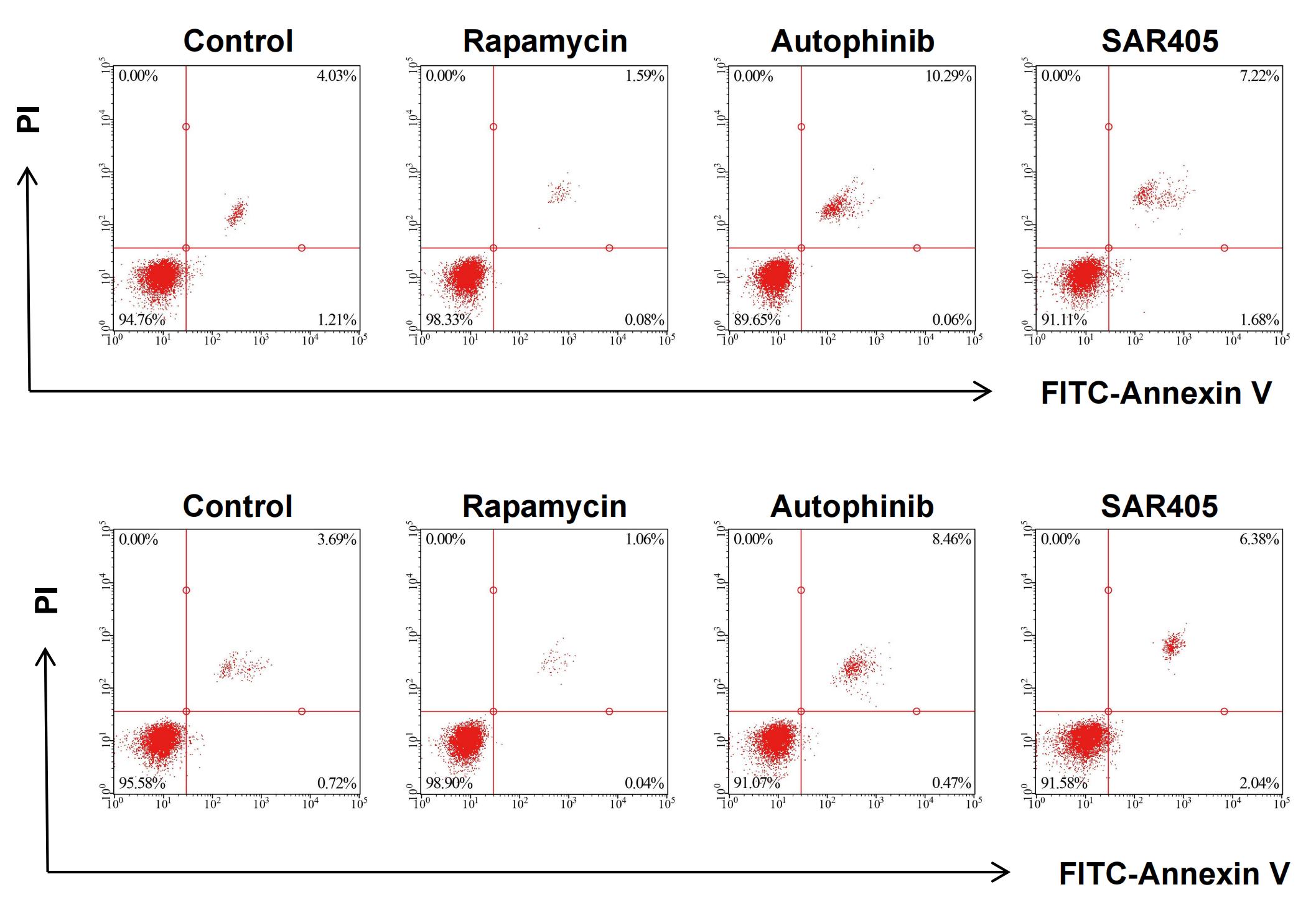

Supplement: Supplementary file 4 — Supplementary Material 4. [file 13046_2026_3713_MOESM4_ESM.jpg]

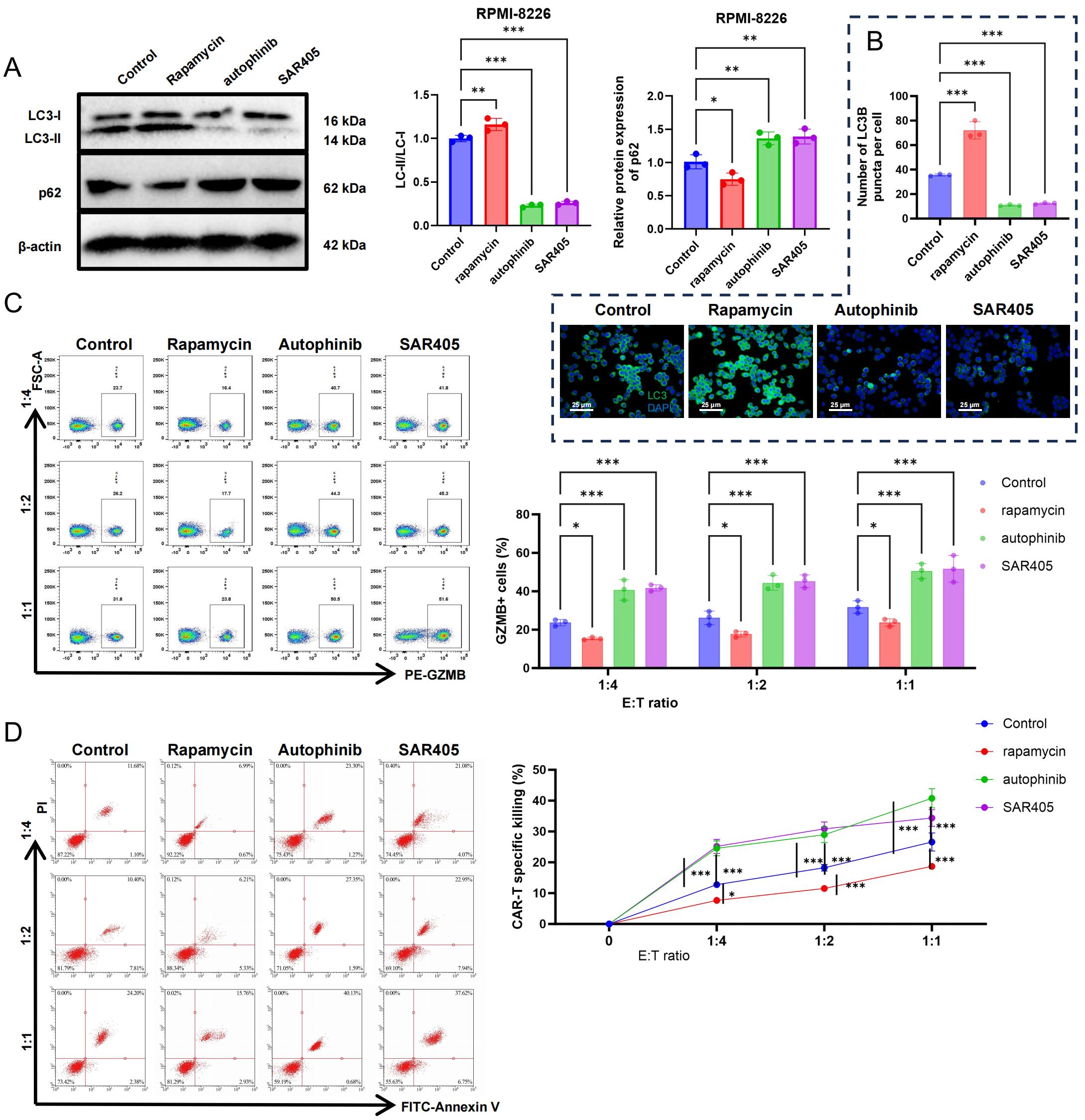

Supplement: Supplementary file 5 — Supplementary Material 5. [file 13046_2026_3713_MOESM5_ESM.jpg]

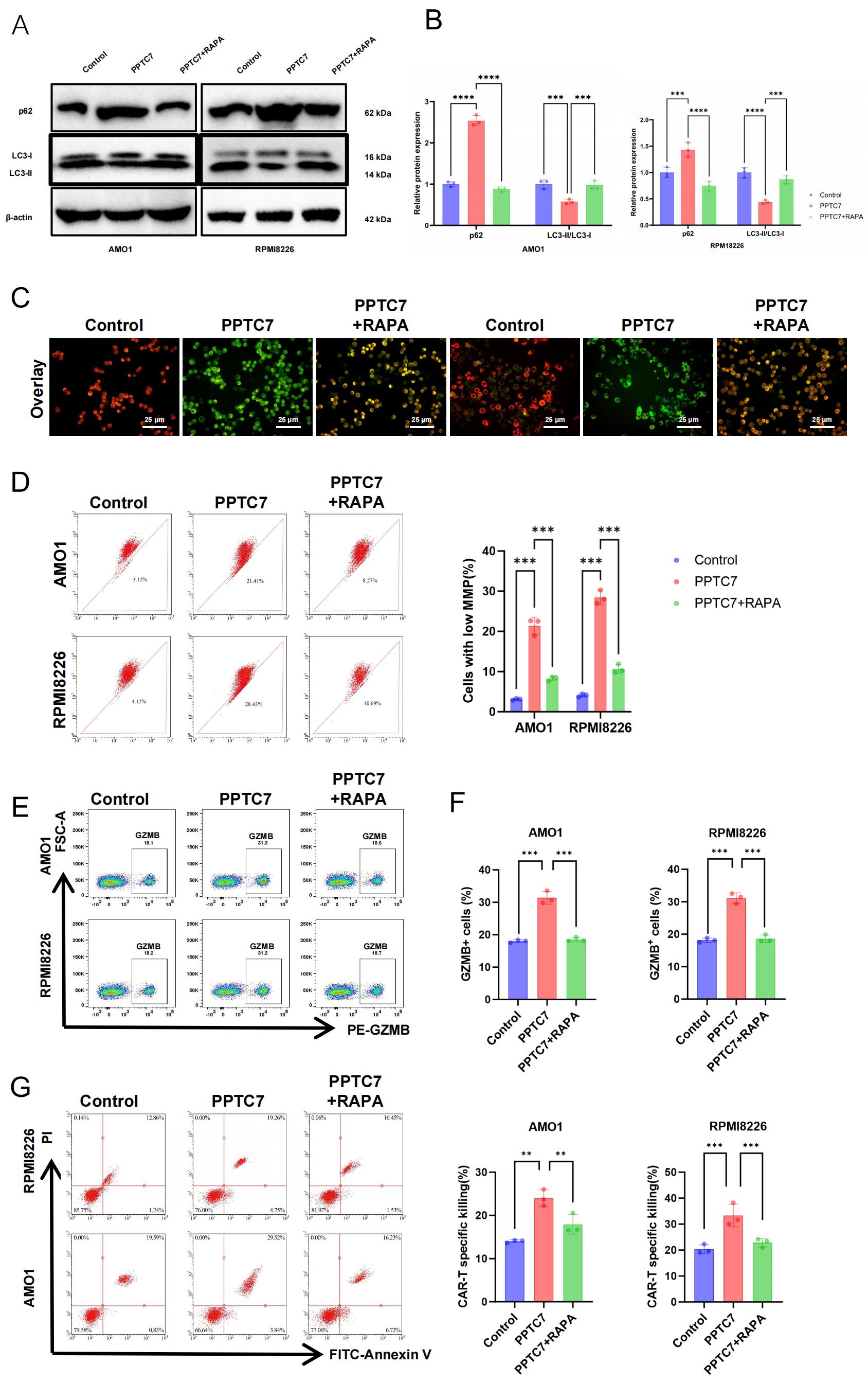

Supplement: Supplementary file 6 — Supplementary Material 6. [file 13046_2026_3713_MOESM6_ESM.jpg]

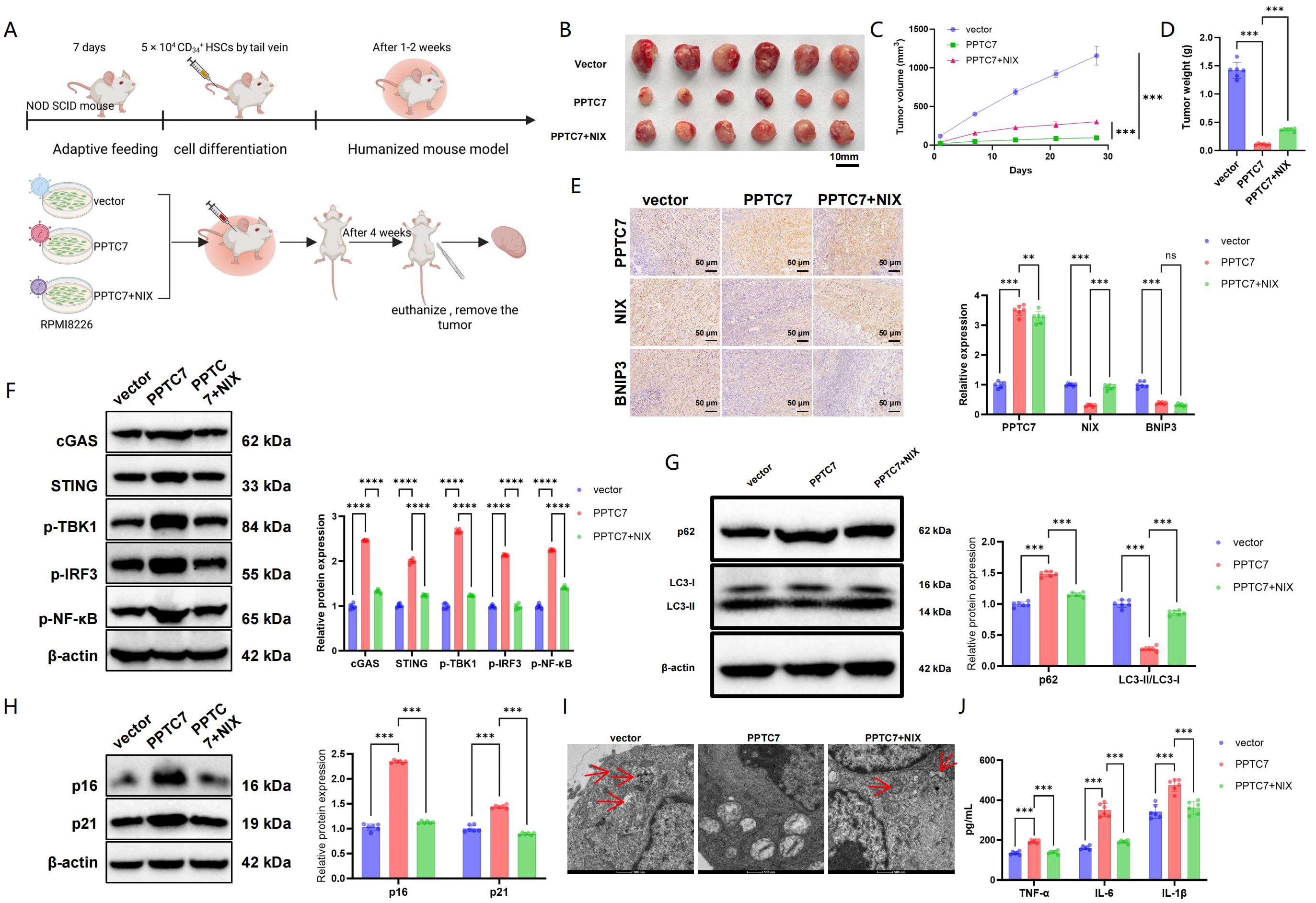

Supplement: Supplementary file 7 — Supplementary Material 7. [file 13046_2026_3713_MOESM7_ESM.jpg]

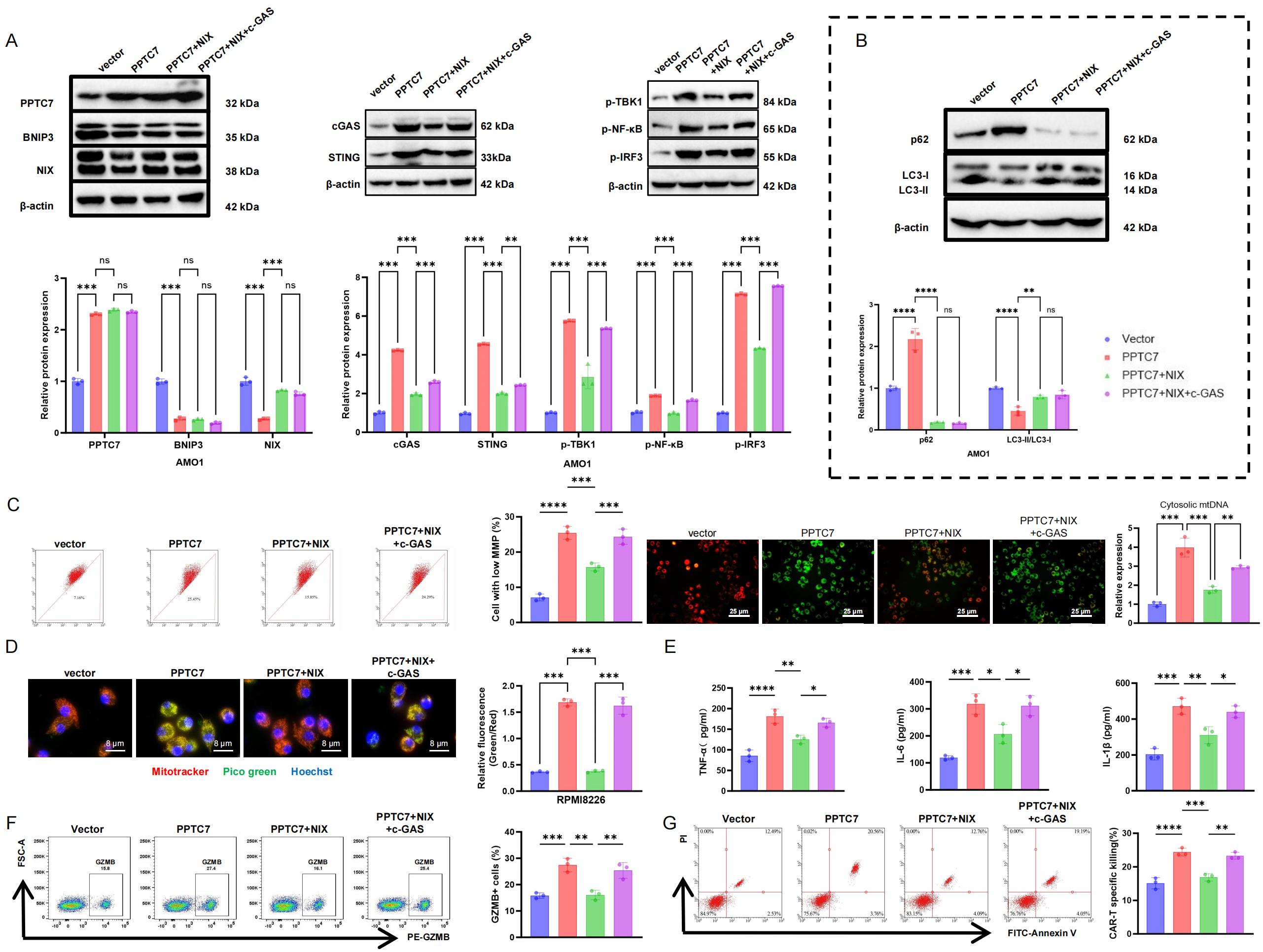

Supplement: Supplementary file 8 — Supplementary Material 8. [file 13046_2026_3713_MOESM8_ESM.jpg]

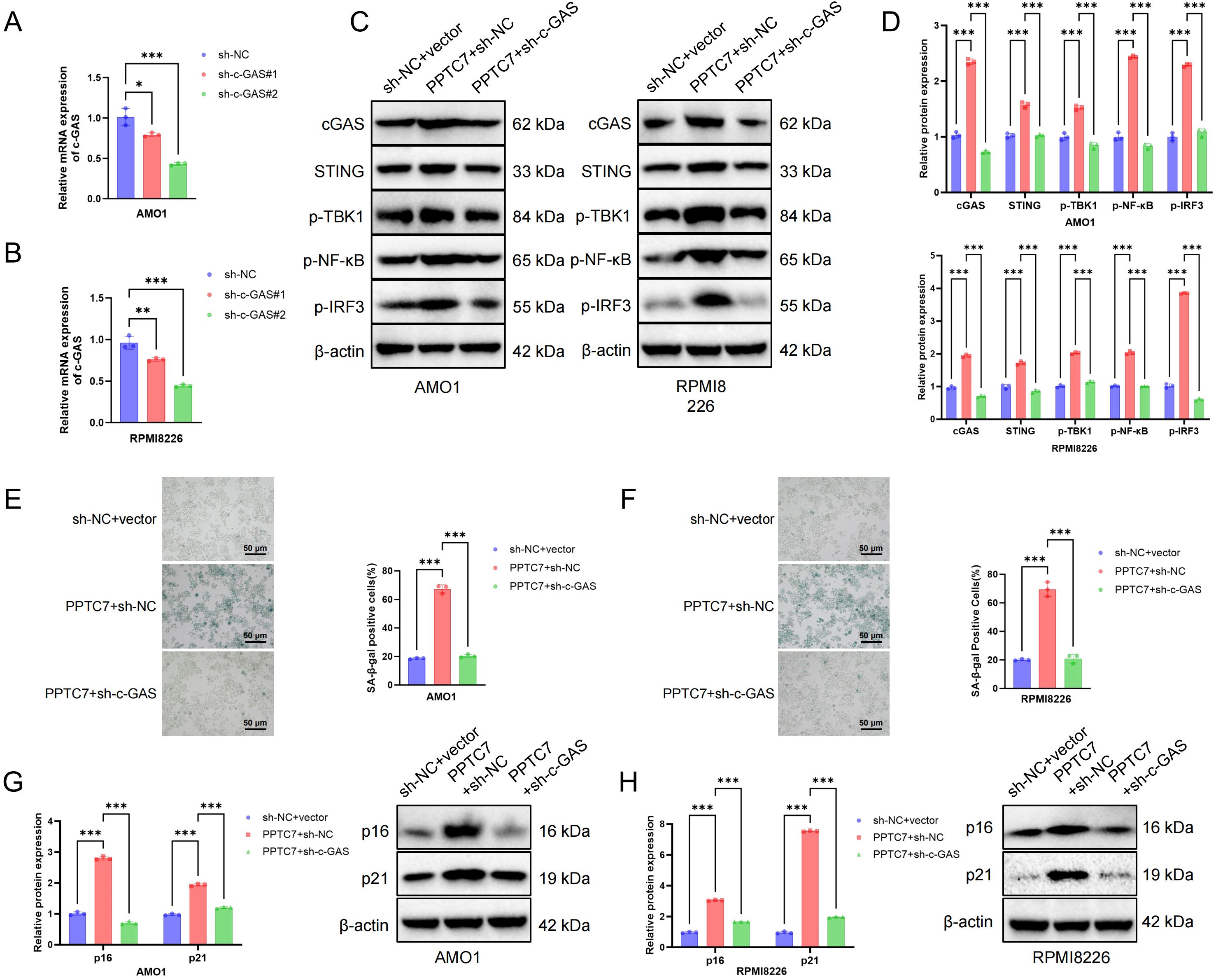

Supplement: Supplementary file 9 — Supplementary Material 9. [file 13046_2026_3713_MOESM9_ESM.jpg]
