# Supplementary material for: The PPTC7/BNIP3/NIX axis induces cGAS/STING-mediated senescence and augments CAR-T efficacy by repressing tumor-intrinsic mitophagy
Source: J Exp Clin Cancer Res. 2026 Apr 18;45:135. doi: 10.1186/s13046-026-03713-7 (PMC13270736; doi:10.1186/s13046-026-03713-7)
Supplement: Supplementary file 10 — Supplementary Material 10. [file 13046_2026_3713_MOESM10_ESM.docx]

**Table S1. shRNA sequence.**

| **Gene ID** | **shRNA** |
| --- | --- |
| sh-PPTC7#1 | CTCAATTCTCAGGGACTTTAA |
| sh-PPTC7#2 | CCACATTTCTTTGCCACTGAT |
| sh-NIX#1 | CAGTCAGAAGAAGAAGTTGTA |
| sh-NIX#2 | GCTAGGCATCTATATTGGAAA |
| sh-BNIP3 #1 | GCCACGTCACTTGTGTTTATT |
| sh-BNIP3#2 | GCCTCGGTTTCTATTTATAAT |
| sh-NC | CCTAAGGTTAAGTCGCCCTCG |
| sh-FBXL4#1 | GCCAGGACTATGTGGAACTTA |
| sh-FBXL4#2 | CGAATTAGTACGCCTTGAATT |
| sh-c-GAS#1 | GATGCTGTCAAAGTTTAGGAA |
| sh-c-GAS#2 | CAACTACGACTAAAGCCATTT |

**Table S2. RT-qPCR primer sequence (human).**

| **Gene ID** | **primer sequence (5'-3')** |
| --- | --- |
| PPTC7-F | CATGAGCTGGCCTATGACCC |
| PPTC7-R | GACGGTGATGTCATCTGGCT |
| FBXL4-F | GGTTCCCTTGCCTTCTCCAT |
| FBXL4-R | TTTGCATGCTGACTTCAGGT |
| BNIP3-F | ACAAGATACCAACAGAGCTTC |
| BNIP3-R | CGACTTGACCAATCCCATATCC |
| NIX-F | CTCTTCCTTTCTCATGTTTTGGC |
| NIX-R | ACTTCACAGGTCACACGC |
| cGAS-F | GAAGGCCTGCGCATTCAAAA |
| cGAS-R | GTGAGAGAAGGATAGCCGCC |
| β-actin-F | AATTTGCGTGTGGCTCCCGAGG |
| β-actin-R | GGATAGCACAGCCTGGATAGCA |
| mtDNA-F | AATCTACCATCCTCCGTGAAACC |
| mtDNA-R | TCAGTTTAGCTACCCCCAAGTTTAA |
| nDNA-F | CTAGCTCATGTGTCAAGACCCTCTT |
| nDNA-R | GCCAGCACGTTTCTCGTT |

Note: F, forward; R, reverse.
